# Oak aging mitigates the sensory impact of smoke taint in Cabernet Sauvignon wine

**DOI:** 10.1038/s41598-026-59592-7

**Published:** 2026-06-30

**Authors:** Lik Xian Lim, Cristina Medina-Plaza, Catherine Routt, Reid Rodriguez, Larry Lerno, Jean-Xavier Guinard, Anita Oberholster

**Affiliations:** 1 Department of Viticulture and Enology, University of California, Davis, Davis, CA 95616 USA; 2 Department of Food Science and Technology, University of California, Davis, Davis, CA 95616 USA

**Keywords:** Smoke taint, Oak aging, Wine quality, Sensory evaluation, Flavor chemistry, Biochemistry, Chemistry, Plant sciences

## Abstract

The wildfires that swept through Napa Valley in 2020 caused an estimated USD$3.7 billion economic loss to the grape and wine industry. The wines made from smoke-impacted grapes are often described as medicinal, smoky, and possessing a retronasal ashtray character. However, matrix differences among varieties and winemaking styles can impact smoke taint expression as well as the synergistic effects among the volatile phenols responsible for these off-flavors. Serial dilutions of non-impacted and impacted (intentionally smoked post-harvest) wines were used to create wines with differing levels of smoke impact for two wine regions (Napa and Lodi) and styles of Cabernet Sauvignon that were fermented and finished in stainless steel (SS) or subsequently aged in new oak barrels for 12 months. Gas chromatography–mass spectrometry (GC–MS) and liquid chromatography-triple quadrupole mass spectrometry (LC-QqQ-MS) were used to quantify free and total volatile phenols (VPs), and individual bound glycosides, respectively. Descriptive analysis using trained judges and Wine Cuality™ evaluations with wine experts were used to evaluate the sensory characteristics and overall quality of the wines, respectively. Generally, barrel-aged wines saw a decrease in the level of “ashy aftertaste” and smoke-related attributes for both Cabernet Sauvignon wines as compared to their stainless-steel counterparts. Wines with high levels of smoke impact received significantly lower quality ratings (*p* < 0.05). Interestingly, wines with low levels of smoke-impact consistently received a slightly higher quality rating though not statistically significant when compared to their non-impacted counterparts. These findings contribute to our understanding of grape smoke exposure at different levels of taint and demonstrate the potential value of barrel aging to mitigate overall smoke perception in wine across these taint levels.

**Supplementary Information:**

The online version contains supplementary material available at https://doi.org/10.1038/s41598-026-59592-7.

## Introduction

In 2020, fires ravaged through winegrowing regions in California, causing large financial losses to the wine industry, estimated to be USD$3.7 billion^1^. Wildfires have become more frequent and severe since the turn of the century^2^. The effects of these fires are a common cause for concern among winemakers and grape growers in wine-producing regions worldwide. When a wildfire occurs, volatile phenols (VPs) are released into the atmosphere. These volatile phenols are absorbed by the grape berry skin and are rapidly glycosylated to form volatile phenol glycosides (VPG) as part of the plant defense mechanism^3–5^. The wines made from these grapes are described as “medicinal”, “campfire”, “smoky”, “burnt” and as having a lingering retronasal “ashtray” aftertaste^6,7^. The grapes, once smoke exposed, have limited options for remediation currently^8^. The impact of wildfires causing smoke taint in wine is thus being felt worldwide, with huge financial implications to producers^1,9^.

The smoky aromas and flavors in wine have been attributed to the family of volatile phenol compounds (guaiacol, *o*-cresol, *p*-cresol, *m*-cresol, 4-methylguaiacol, 4-methylsyringol, syringol, and 4-ethylguaiacol) and volatile phenol glycosides (syringol gentiobioside, methylsyringol gentiobioside, cresol rutinoside, guaiacol rutinoside, methylsyringol rutinoside, and phenol rutinoside)^10,11^. Both volatile phenols (VPs) and volatile phenol glycosides (VPGs) contribute to smoky flavors and aromas through subthreshold interactions, even when any individual compound is present below its sensory threshold. VPGs, in particular, contribute to the smoke flavor and the retronasal “ashy” aftertaste via in-mouth hydrolysis during wine tasting^5,12,13^. These compounds occur naturally in non-smoke-impacted grapes and wines, making it essential to understand their background levels when assessing potential smoke exposure^14,15^. The central question currently being studied by research groups worldwide is determining the concentrations of smoke marker compounds in wines that cause a perceptible smoky flavor and/or ashy aftertaste.

Some smoky aromas and compounds are naturally present in foods, wines and other beverages and are often considered desirable^16–18^. In commercially produced wines, aging in toasted oak barrels can increase guaiacol and other volatile phenols, imparting toasty and smoky flavor notes^19^. Similar smoky characteristics are also appreciated in certain spirits, such as peated Scotch whiskies and Mezcals^18,20^.

Wine is a particularly complex matrix composed of water, alcohol, organic acids, amino acids, phenols, tannins, sugars, and numerous other compounds that together define its distinctive character^21,22^. These components are influenced by abiotic factors such as vineyard microclimate and other terroir elements^23,24^. In California, two primary styles of Cabernet Sauvignon dominate: a full-bodied style from Napa Valley, representing the high-end luxury wine market, and a medium-bodied style from the Central Valley, representing the mass-market segment^25^. Notably, Napa Valley accounts for only about 5% of California’s wine production by volume, while the Central Valley produces approximately 70%, yet Napa Valley accounts for more than 50% of the revenue share for the entire California wine industry^26–28^. In the current study, the impact of oak aging on smoke taint expression was examined in both styles of Cabernet Sauvignon wine.

There have been several strategies tested and used to mitigate the impact of smoke-taint in wine. Vineyard treatments include barrier sprays or activated carbon fabric in the vineyard to prevent the uptake of smoke compounds into the grape^29–32^. Winemaking strategies include reducing skin contact time with the juice to prevent the extraction of the smoke compounds, oak additions, or having slightly higher amounts of residual sugar to inhibit in-mouth release of the ashy aftertaste^33–36^. Post-fermentation remediation strategies such as solid-phase adsorption, reverse osmosis, and fining agents (e.g., activated carbon) have been applied to smoke-impacted wines^37–39^. However, these methods are generally non-selective and often remove desirable flavor and aroma compounds along with the smoke-related volatiles^8^. Notably, most studies on smoke-taint mitigation have not examined the effects of barrel aging, despite its widespread commercial use, particularly in premium wine production regions such as Napa Valley^40–42^. Oak barrels are commonly employed to enhance wine flavor and structure by softening tannins and imparting oak-derived aromas. Depending on the degree of toast, barrels can contribute volatile phenols (e.g., guaiacol, cresols) while also potentially adsorbing volatile phenol glycosides, as hypothesized by Ristic et al.^35^. Such interactions may reduce smoke expression, add complexity, and improve overall wine acceptability as a result^43^.

The central principle in sensory science is that the test method must be aligned with the research objective^44^. In this study, the first objective was to characterize the sensory profile of the wines, for which a modified descriptive analysis was employed^45^. The second objective was to evaluate the overall sensory quality of the wines, for which we turned to industry experts and had them use our newly developed Wine Cuality™ method^46^. The method has experts provide overall quality ratings on a 100-point scale, similar to current wine quality ratings by wine experts. Then the experts rate the adequacy of key sensory attribute intensities on just-about-right (JAR) scales, describe the sensory attributes of the wines from a check-all-that-apply (CATA) list of sensory and holistic attributes, and finally provide comments about what specifically they liked or disliked about the wine. A statistical suite that includes Internal and External Quality Mapping (a PCA of the quality ratings across the wines and a regression of expert quality ratings onto a sensory map) as described in Delgado and Guinard^47^, a correspondence analysis of the CATA selections, penalty analysis relating quality ratings to JAR ratings, a penalty-lift analysis relating quality ratings to CATA selections, and word analyses of CATA selections and open comments allow for a thorough deconstruction and justification of the overall quality ratings. The measurement of acceptance by consumers is the other piece of a comprehensive sensory study, and it is pending. Though it is important to note that some research groups have investigated the consumer acceptance of smoke-tainted wines in other studies^48,49^. Here, the Wine Cuality™ method was applied to compare stainless-steel and barrel-aged wines separately, allowing for a more holistic understanding of smoke impact across wine styles^50–52^. Those quality ratings from the experts were then related to the descriptive analysis ratings by the trained panelists, where quality could be regressed onto the descriptive data to further understand positive and negative drivers of quality for this set of stainless-steel and oak-aged, smoke-taint free or smoke-tainted Cabernet Sauvignon wines.

In this study, we investigated the smoke taint mitigating potential of oak barrel aging in Cabernet Sauvignon wine with a research design that combined post-harvest smoke exposure on two styles of Cabernet Sauvignon at different winemaking stages (stainless-steel and oak-barrel aged) using both descriptive analysis by trained panellists and quality assessment by wine experts. We tested the hypothesis that oak aging can mitigate the impact of smoke taint by contributing (desirable) flavors and mouthfeel that would reduce the perceived intensity of smoke-associated defects and add to the wine’s complexity.

## Materials and methods

### Grapes

In the 2022 harvest, Cabernet Sauvignon wine grapes were hand-harvested in the Napa Valley (NV), USA, and in Lodi (LD), Central Valley, USA, and delivered to the UC Davis Teaching and Research Winery (Davis, CA, USA) for processing. All grapes were donated by and collected with permission from commercially farmed vineyards. The Napa and Lodi origins for the grapes were selected to represent different viticultural areas, climates and terroirs, and to be made into a premium, full-bodied style for the Napa Cabernet Sauvignon and into a medium-body style for the Lodi Cabernet Sauvignon. Each lot of grapes was split into two treatments, non-smoked (NST) and smoked (ST). The ST grapes were intentionally smoked in a purpose-built smoking tent^53^. The grapes were transferred into 226 Kilogram macrobins, MacroBin 14-FV (IPL Macro, Fairfield, USA) which had holes on the sides and bottom to promote the flow of smoke through the grapes. These macrobins were then situated in a purpose-built smoking tent 12 ft × 5 ft × 6 ft, covered with six-mil polyethene sheeting (Frost King & Thermwell Products Co., Inc., Mahwah, NJ, USA). The grapes were smoked using two Z Grills pellet smokers (Z Grills Inc., Ontario, CA, USA) at a rate of 200 g/hour of hickory wood pellets from Traeger (Traeger, Salt Lake City, UT, USA) per smoker to give a wine of maximum smoke impact. The amount of grapes for each smoking session and the times are summarized in Table 1. There were two different lots of Cabernet Sauvignon grapes from Napa Valley (NV_A and NV_B) and one lot of Cabernet Sauvignon from Lodi. These grapes were smoked independently. After smoking, the grapes were allowed to sit at room temperature between 18 and 24 °C for 8 hours before undergoing winemaking. All grapes for this study were donated by commercially farmed vineyards.

**Table 1:** Summary of grape variety, location, smoking time, weight, and basic grape chemical composition.

Wine code	Varietal, location	Smoking time (hours)	Weight (lbs)	Brix (°)	pH	Titratable acidity (g/L)	Malic acid (mg/L)	Yeast assimilable nitrogen (mg/L)
NV_A_NST	Cabernet Sauvignon, Napa Valley	NA	900	24.45 ± 0.21	3.66 ± 0.01	5.57 ± 0.04	1450.00 ± 28.28	136.50 ± 3.54
NV_A_ST	Cabernet Sauvignon, Napa Valley	2	900	25.10 ± 0.28	3.79 ± 0.01	5.36 ± 0.01	1470.00 ± 14.14	135.50 ± 0.71
NV_B_NST	Cabernet Sauvignon, Napa Valley	NA	1200	27.37 ± 0.06	3.96 ± 0.04	5.34 ± 0.19	1490.00 ± 36.06	117.67 ± 2.89
NV_B_ST	Cabernet Sauvignon, Napa Valley	2.5	1200	28.00 ± 0.20	4.04 ± 0.00	5.53 ± 0.05	1556.67 ± 41.63	129.33 ± 0.58
LD_NST	Cabernet Sauvignon, Lodi	NA	2500	25.75 ± 0.07	4.22 ± 0.03	4.52 ± 0.11	2005.00 ± 7.07	107.00 ± 2.83
LD_ST	Cabernet Sauvignon, Lodi	5	2500	24.90 ± 0.00	4.22 ± 0.00	4.77 ± 0.02	2205.00 ± 63.64	137.00 ± 2.83

Mean values ± standard deviation (n = 3 for NV_B, and n = 2 for NV_A and Lodi) for brix, pH, titratable acidity, malic acid, and yeast assimilable nitrogen. Means were calculated using analytical replicates.

### Winemaking

#### Premium, full-bodied Cabernet Sauvignon, Napa Valley

Generally, NST and ST grapes were destemmed and crushed using a Bucher Vaslin Delta E2 destemmer and crusher (Bucher Vaslin North America, Santa Rosa, CA, USA) into 200 L stainless-steel vessels, approximately 180 kg per vessel, for two fermentation replicates(n = 2) in NV_A and three fermentation (n = 3) replicates for NV_B. 50 mg/L of sulfur dioxide (SO_2_) was added using a 15% potassium metabisulfite solution, K_2_S_2_O_5_ (Laffort, Petaluma, CA, USA). Additions were made to each vessel to adjust the titratable acidity (TA) to 6.0 g/L using tartaric acid (CalSoda, Rohnert Park, CA, USA), and yeast assimilable nitrogen (YAN) to 250 mg/L using diammonium phosphate, (NH_4_)_2_HPO_4_, (Laffort, Petaluma, CA, USA). For batch NV_B, for 3 fermentation replicates (n = 3), Brix was adjusted to 25° Brix using acidified (6.0 g/L tartaric acid) water. The wine was cold-soaked at 15 °C for 24 h. The next day, the vessels were heated to 25 °C and inoculated with *Saccharomyces cerevisiae* strain EC1118 (Lallemand, Montreal, Canada) at 0.264 g/L using the rehydration procedure from the manufacturer. Fermentation was controlled at 25 °C, with cap-management set to one tank volume pump-over every 12 h. After 10 days of maceration, wines were pressed and inoculated with *Oenococcus oeni* VP41 to induce malolactic fermentation (MLF) (Lallemand, Montreal, Canada). MLF was checked weekly until complete, when malic acid levels were below 200 mg/L as determined enzymatically. Wines were racked off lees and SO_2_ adjusted to 35 mg/L free SO_2_. Fermentation replicates were tasted and chemically analyzed to ensure no differences in the wines before they were combined based on their treatment, NST or ST.

The wine from each treatment (NST and ST) was split into two lots: stainless-steel (SS) and oak barrel-aged (BL). SS wines (120 L) were stored in stainless-steel vessels for 12 months (n = 1, aging replicate), whereas BL wines were aged for 12 months in two (n = 2 aging replicate) new 55 Liter, Demptos, Bordeaux, Cooper Select -TG, Medium plus toast French oak barrels from Demptos Napa Cooperage (Napa, CA, USA). SO_2_ was checked and adjusted monthly to 35 mg/L free SO_2_. The barrels were topped monthly with their respective treatments.

#### Medium-bodied Cabernet Sauvignon, Lodi

The grapes (1150 kg each), NST and ST, were destemmed into a 2000 kg stainless-steel fermentation tank. 50 mg/L of free SO_2_ was added using potassium metabisulfite, K_2_S_2_O_5_. Yeast assimilable nitrogen (YAN) was adjusted to 250 mg/L as needed using diammonium phosphate ((NH_4_)_2_HPO_4_), and TA was adjusted to 7.0 g/L with tartaric acid. This was done to account for the higher pH and higher malic acid levels. Ultimately, the goal was to have a wine that was similar in alcohol, residual sugar and acidity, to the Napa Valley Cabernet Sauvignon. *Saccharomyces cerevisiae* strain EC1118 was used for the fermentation. Cap-management was set to one tank volume every 12 h. After 11 days of maceration, wines were pressed and inoculated with *Oenococcus oeni* VP41 to induce malolactic fermentation (MLF). MLF was complete when malic acid levels fell below 200 mg/L, measured enzymatically. Wines were racked off lees and SO_2_ adjusted to 35 mg/L free SO_2_. These wines went through rough filtration via a plate and frame filter using FibraFix AF 100 depth filter sheets (Filtrox, St. Gallen, Switzerland) with a nominal pore size of 0.6–1.5 μm. Subsequently, wines were sterile filtered using in-line ALpHA MF0.8-1F6RS and SteriLUX VMH0.4-1F6RS filters (Meissner, Camarillo, CA).

Each NST wine and ST wine was then split into two batches: stainless steel and oak barrel. The wines were aged for 12 months in new 226 L TW Boswell, Bordeaux, Elevate, medium plus toast French oak from Cooperages 1912 (Napa, CA, USA) or held in stainless-steel vessels. SO_2_ was checked and adjusted monthly to 35 mg/L free SO_2_. The barrels were topped monthly with their respective treatments.

#### Blending

After 12 months, a bench tasting by the research team (n = 3 tasters) was conducted to blend the wines. The research team is highly proficient in evaluating the wines and their smoke characteristics. In the bench tasting, the stainless-steel ST wine was diluted by a factor of two with the NST wine until there were no more smoky or ashy characteristics present. One further dilution was then made. In total, we made seven wines in the dilution scheme, labeled as L0, L1, L2, L3, L4, L5, and ST, without replication, where L0 (NST)was the non-smoke-impacted wine and ST is the fully smoke-impacted wine. A summary of the levels can be found in the decoder shown in Table 2. For the oak-aged wines, the levels were matched to those of the stainless-steel wines to assess the effectiveness of oak aging for smoke taint mitigation.

**Table 2:** Decoder for the Napa and Lodi Cabernet Sauvignon wine blends, the treatments, and the subsequent identifiers.

Location	Treatment	Amount smoke impacted wine in blend	Identifier
Full Bodied, Napa Valley			
Napa Valley	Stainless steel	0.00%	NV_SS_L0
Napa Valley	Stainless steel	1.56%	NV_SS_L1
Napa Valley	Stainless steel	3.12%	NV_SS_L2
Napa Valley	Stainless steel	6.25%	NV_SS_L3
Napa Valley	Stainless steel	12.50%	NV_SS_L4
Napa Valley	Stainless steel	25.00%	NV_SS_L5
Napa Valley	Stainless steel	100.00%	NV_SS_ST
Napa Valley	Oak barrel	0.00%	NV_BL_L0
Napa Valley	Oak barrel	1.56%	NV_BL_L1
Napa Valley	Oak barrel	3.12%	NV_BL_L2
Napa Valley	Oak barrel	6.25%	NV_BL_L3
Napa Valley	Oak barrel	12.50%	NV_BL_L4
Napa Valley	Oak barrel	25.00%	NV_BL_L5
Napa Valley	Oak barrel	100.00%	NV_BL_ST
Medium Bodied, Lodi, Central Valley			
Lodi	Stainless steel	0.00%	LD_SS_L0
Lodi	Stainless steel	1.56%	LD_SS_L1
Lodi	Stainless steel	3.12%	LD_SS_L2
Lodi	Stainless steel	6.25%	LD_SS_L3
Lodi	Stainless steel	12.50%	LD_SS_L4
Lodi	Stainless steel	25.00%	LD_SS_L5
Lodi	Stainless steel	100.00%	LD_SS_ST
Lodi	Oak barrel	0.00%	LD_BL_L0
Lodi	Oak barrel	1.56%	LD_BL_L1
Lodi	Oak barrel	3.12%	LD_BL_L2
Lodi	Oak barrel	6.25%	LD_BL_L3
Lodi	Oak barrel	12.50%	LD_BL_L4
Lodi	Oak barrel	25.00%	LD_BL_L5
Lodi	Oak barrel	100.00%	LD_BL_ST

The blending dilution was done without replication due to limited availability of wine required to generate additional blending replicates. In addition, the dilution involved direct post fermentation and post aging blending of finished wines, where compositional differences between replicate preparations are expected to be minimal under controlled conditions. Replication would also have tripled the number of samples required for sensory evaluation, which would have exceeded practical limits of doing a sensory test. Therefore, the lack of replication is not expected to influence the overall interpretation of treatment (blending) effects.

### Chemical analysis

#### Free and acid-labile (total) volatile phenols.

Liquid–liquid extraction (LLE) with pentane-ethyl acetate (1:1) was used to quantify guaiacol, 4-methylguaiacol, *o*-cresol, phenol, 4-ethylguaiacol, *p*-cresol, *m*-cresol, 2,3-dimethoxyphenol, 4-ethylphenol, syringol, and 4-methylsyringol in samples. For the acid-labile VPs (total VPs), harsh acid-hydrolysis at pH 1, 100 °C for 1 h was first done before LLE. A detailed description of the method can be found in Oberholster et al.^34^. An Agilent 7890A gas chromatograph coupled to an Agilent 7000B triple quadrupole mass spectrometer with an MPS 2 autosampler (Gerstel, Inc., Linthicum, MD) was used as described in Lim et al.^45^. Analytical grade chemicals 4-methylsyringol, 4-ethylphenol, 4-ethylguaicol, and hydrochloric acid (HCl) were purchased from Sigma-Aldrich (St. Louis, MO, USA). Guaiacol, *o*-cresol, *m*-cresol, and *p*-cresol were obtained from TCI America (Portland, OR, USA). Syringol and 4-methylsyringol were obtained from Alfa Aesar (Tewksbury, MA, USA). The deuterated standards (d3-guaiacol, d3-4-methylguaiacol, d7-*o*-cresol, d7-*p*-cresol, d7-*m*-cresol, d5-4-ethylguaiacol, and d4-4-ethylphenol) were obtained from CDN Isotopes (Pointe-Claire, QC, Canada) and d6-syringol were obtained from EPTES (Vevey, Switzerland). HPLC grade solvents, ethyl acetate, pentane, and ethanol, were obtained from Sigma-Aldrich (St. Louis, MO, USA).

#### Volatile phenol glycoside (individual bound) analysis

The wine samples were first concentrated using solid phase extraction (SPE) as per the method in Oberholster et al.^34^. The samples were then analyzed using liquid chromatography tandem mass spectrometry (LC–MS/MS) on an Agilent 1290 Infinity UHPLC (Agilent Technologies, Santa Clara, CA) equipped with a binary pump, temperature controlled column compartment, and temperature-controlled autosampler. LC–MS/MS as described in Lim et al.^45^. The deuterated VP glycoside standards were obtained from CDN Isotopes Inc. (Quebec, Canada), Toronto Research Chemicals (Toronto, Canada), and EPTES (Vevey, Switzerland). Calibration curves were constructed for all glycosides with deuterated VP glycosides as internal standards.

Quantitative analysis of the LC–MS/MS data and GC–MS data was conducted using the Mass Hunter Workstation software suite (version B.09.00, Agilent Technologies, Santa Clara, CA, USA).

#### Wine analysis

The chemical composition of the wines was analyzed daily during the descriptive analysis. Titratable acidity (tartaric acid equivalents) and pH (8.2 endpoint) were measured using a Mettler-Toledo DL50 titrator (Mettler-Toledo Inc., Columbus, OH, USA) and Orion 5-star pH meter (Thermo Fisher Scientific, Waltham, MA, USA), respectively. The ethanol content % (v/v) was measured using an alcohol analyzer (Anton Parr, Ashland, VA, USA). Malic acid, residual sugar (RS), and acetic acid (AA) were determined by enzymatic analysis using a Biosystems SPICA analyzer (Admeo Inc., Napa, CA, USA).

### Sensory evaluation

#### Descriptive panel recruitment

Sensory panelists were recruited based on their availability, interest, consumption frequency of red wine (at least once a week), and previous experience in tasting red wines and in descriptive analysis. Panelists were screened for their ability to detect smoke using a series of ashy standard dilutions^45^.

Sensory analysis was conducted in accordance with the guidelines of the Declaration of Helsinki and was approved by the Institutional Review Board (IRB) of the University of California, Davis – UC Davis IRB Protocol 1288072-1. Informed consent was obtained from all participants.

#### Expert recruitment for the Wine Cuality™ assessment

An expert panel consisting of winemakers, enologists, wine academics, sommeliers, and wine buyers was recruited to evaluate the sensory quality of the wines. All panelists tasted wines as part of their daily jobs. That component of the research also was conducted in accordance with the guidelines of the Declaration of Helsinki and was approved by the Institutional Review Board (IRB) of the University of California, Davis – UC Davis IRB Protocol 1288072-2. Informed consent was obtained from all participants.

#### Modified descriptive analysis

A modified descriptive analysis method was used to evaluate the wines. A decision was made to exclude the L1 wine from the DA, as it was deemed too similar to the L0 wine through bench testing. The exact proportion of smoke-tainted wine in each dilution level is provided in Table 2. Although L1 was excluded from the DA due to its sensory similarity to L0/NST during bench testing, it was retained in the Wine Cuality™ assessment to allow expert evaluation of subtle quality changes across the full smoke-impact dilution series.

The DA panel reached a consensus on seventeen aroma descriptors and standards (7 smoke-related standards), six mouthfeel/taste descriptors and standards, and one ashy aftertaste and standard, which were sufficient to describe the wines presented to them (Supplementary Table S1). The ashy standard was prepared as described in Lim et al.^45^. The panelists underwent four training sessions where they were familiarized with the data collection software used, RedJade (Redwood City, CA, USA), and practiced rating the intensities of the attributes in the scorecard. The participants’ performance was checked at each training session, and where needed, additional training was provided. Panelists were all eligible to participate in the DA panels, Napa Cabernet Sauvignon panel (n = 11), and Lodi Cabernet Sauvignon panel (n = 12). One panelist did not complete the Napa Cabernet Sauvignon panel, and two panelists did not complete the Lodi Cabernet Sauvignon panel. Each panel had 10 judges, 4 males and 6 females, ages 22–64 years for the Napa CS panel, and 3 males and 7 females, ages 22–64 years for the Lodi CS panel.

The DA panels for the Napa and Lodi Cabernet Sauvignon wines ran sequentially, one after another. There was a total of six days of evaluations for each of the DA panels. Panelists evaluated six wines each day for both DA panels. The descriptive analysis training was conducted in the Silverado Vineyards Sensory Theater at the Robert Mondavi Institute for Wine and Food Science, at the University of California, Davis (Davis, CA, USA). The sensory theater is a pressure-positive room that could hold all panelists at once and allowed for effective interaction and discussion during term generation and training. Formal evaluations were held in positive-pressure individual red-light booths. Approximately 30 mL of wine was served in a black Riedel wine glasses (item number #0446 Zinfandel/Riesling, Riedel Crystal of America, Edison, NJ, USA). Before evaluations each day, participants had to complete an aroma quiz to identify the aromas of the reference standards. A 3-digit blinding code was assigned to each wine. All wines were tasted in triplicate, in a Williams Latin square design. Participants were given a code to log in to RedJade to access the survey. A 15 cm unstructured line scale, with anchors at 0% (not present), 10% (low presence), 90% (very intense), 100% (max intensity), was used. Participants first assessed the aroma of the wine without tasting, then took a sip of the wine, expectorated, and evaluated the taste and mouthfeel attributes. Finally, they would take a second sip and evaluate the ashy retronasal aftertaste over a thirty-second period after expectoration, where the highest level of the ashy aftertaste character was rated. There was an enforced 2-min wait between samples to minimize carryover effects^34,54,55^. Panelists were provided a glucose solution (4 g/L), unsalted saltine crackers, and plain bottled water (Kirkland Signature, Costco Wholesale, Seattle, WA) to rinse their mouths and cleanse their palate between samples.

#### Wine Cuality™ analysis

The Wine Cuality^™^ method was used to evaluate the sensory quality of the wines with wine experts. Wine Cuality consists of a 100-point score, just-about-right (JAR) scaling for select attributes, check-all-that-apply (CATA), and open comments regarding specific likes and dislikes. We developed, tested and validated the method as described in Lim et al.^46^. A total of 43 wine experts (19 men, 23 women, ages 25-65 years old) rated the quality of the full dilution set of each wine for both the stainless-steel and oak-aged wines for both styles of Cabernet Sauvignon as part of the method validation research.

### Statistical analysis

All statistical analyses were conducted using R, version 4.3.3 “Angel Food Cake” (R Core Team, 2024)^56^, and R Studio Version 2023.12.1 + 402 (RStudio Inc., 2024), at a significance level of α = 0.05.

#### Descriptive data analysis

DA data was exported from RedJade, which automatically converted the position on the 15 cm line scale to a score between 0 and 100 for each attribute. For each wine style, Napa and Lodi Cabernet Sauvignon, and treatment type, stainless-steel or barrel-aged treatment, a three-factor multivariate analysis of variance (MANOVA) for judge, product, and replicate factors was performed across all attributes. Next, a three-factor univariate pseudo-mixed model analysis of variance (ANOVA) with two-way interactions between judges, products, and replicates was used to identify significant DA attributes^57^. Product was treated as the fixed effect, while judge and replicate effects were included to account for panelist variability and repeated evaluations. Product effects were tested using the judge-by-product and replicate-by-product interaction terms as appropriate error terms.

A mixed-effects model was also evaluated, with judge treated as a random effect to account for panelist-to-panelist variability and repeated sensory ratings. The mixed-effects model produced the same overall interpretation of product, treatment, and dilution effects as the pseudo-mixed ANOVA. Therefore, the pseudo-mixed ANOVA approach was retained for the final analysis.

Fisher’s Least Significant Difference (LSD) was calculated only for attributes with significant product effects in the pseudo-mixed ANOVA. LSD values were calculated separately for Napa stainless-steel, Napa barrel-aged, Lodi stainless-steel, and Lodi barrel-aged wines. Mean score plots were then used to visualize DA attribute differences between stainless-steel and barrel-aged treatments within each wine type.

Principal component analysis (PCA) was used to evaluate and visualize how the wines differed across significant attributes for both the stainless-steel and oak barrel treatments. To assess how the wines differed across both chemical and descriptive sensory data, a multiple factor analysis (MFA) was used^58^.

#### Wine quality data analysis

The Cuality™ Statistics suite of internal quality mapping and clustering, penalty analysis, and penalty/lift analysis was used to analyze the 100-point quality scores, JAR ratings, and CATA selections^46,47,59^. Here, the most important measure was the 100-point quality score, which was used to assess how smoke taint and oak mitigation thereof affected the perceived quality of the wines.

#### Relating quality ratings to descriptive analysis data

First, the quality scores were overlaid on the PCA generated previously to visualize overall wine quality and facilitate the interpretation of how these scores relate to the descriptive sensory attributes^47^. We note again that wine L1 was excluded from this analysis. Second, the quality scores were related to the mean scores of the significant DA attributes for the barrel and stainless-steel wine aging treatments, using a mean score plot for the DA attributes and line-plot for the quality scores.

Correlation and regression analysis were performed to relate the sum of the chemical compounds (VPG, FVP and TVP), ashy rating, and overall quality score across the smoke impact dilution levels. For each regression, six data points were used, corresponding to the mean values for L0, L2, L3, L4, L5, and ST; L1 was not included because it was excluded from the descriptive analysis. Regressions were performed separately for each wine origin and aging treatment combination, Napa stainless-steel, Napa barrel-aged, Lodi stainless-steel, and Lodi barrel-aged. Therefore, these analyses describe within-series relationships across ordered dilution levels, rather than independent predictive relationships across unrelated wine samples. Each regression was based on six mean values from an ordered dilution series, the analysis was interpreted descriptively, and R^2^ values were used only to summarize within-series trends rather than to establish predictive model performance.

## Results

### Chemical composition, free and total volatile phenols, and individual bound glycosides

The wines did not show any significant differences in their basic chemical composition across ethanol content, pH, titratable acidity, residual sugar, malic acid, and acetic acid for the Napa and Lodi Cabernet Sauvignon wines, respectively (Supplementary Table S2). This meant that the main chemical differences among the wines would be the free VPs, total VPs, and volatile phenol glycosides, if any, which was the goal of this study’s design.

**Fig. 1:**
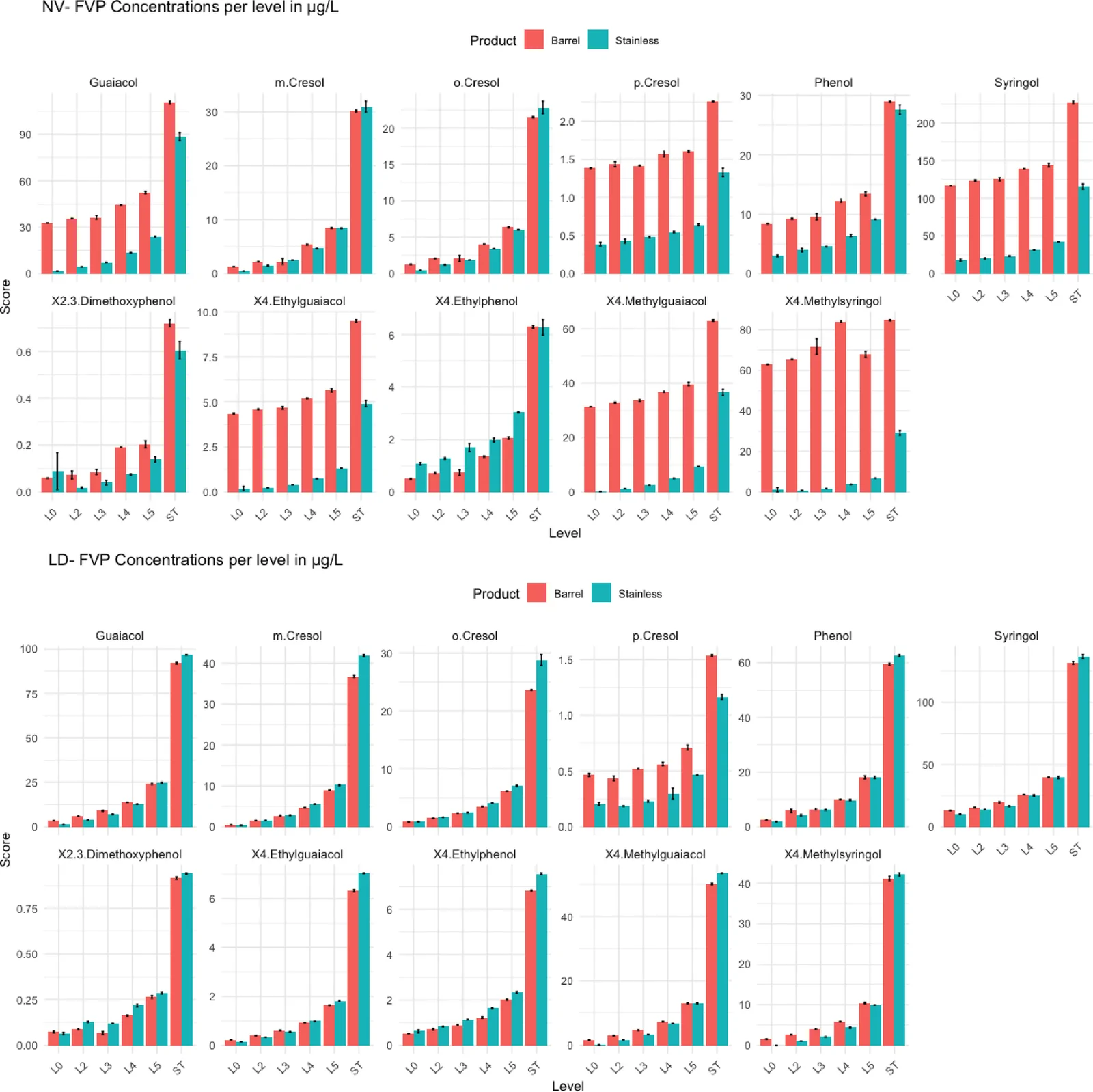
Mean free volatile phenol concentrations (μg/L) per smoke taint level, with standard errors, comparing the barrel-aged and stainless-steel wines for the Napa Cabernet Sauvignon (NV, top) and Lodi Cabernet Sauvignon (LD, bottom).

**Fig. 2:**
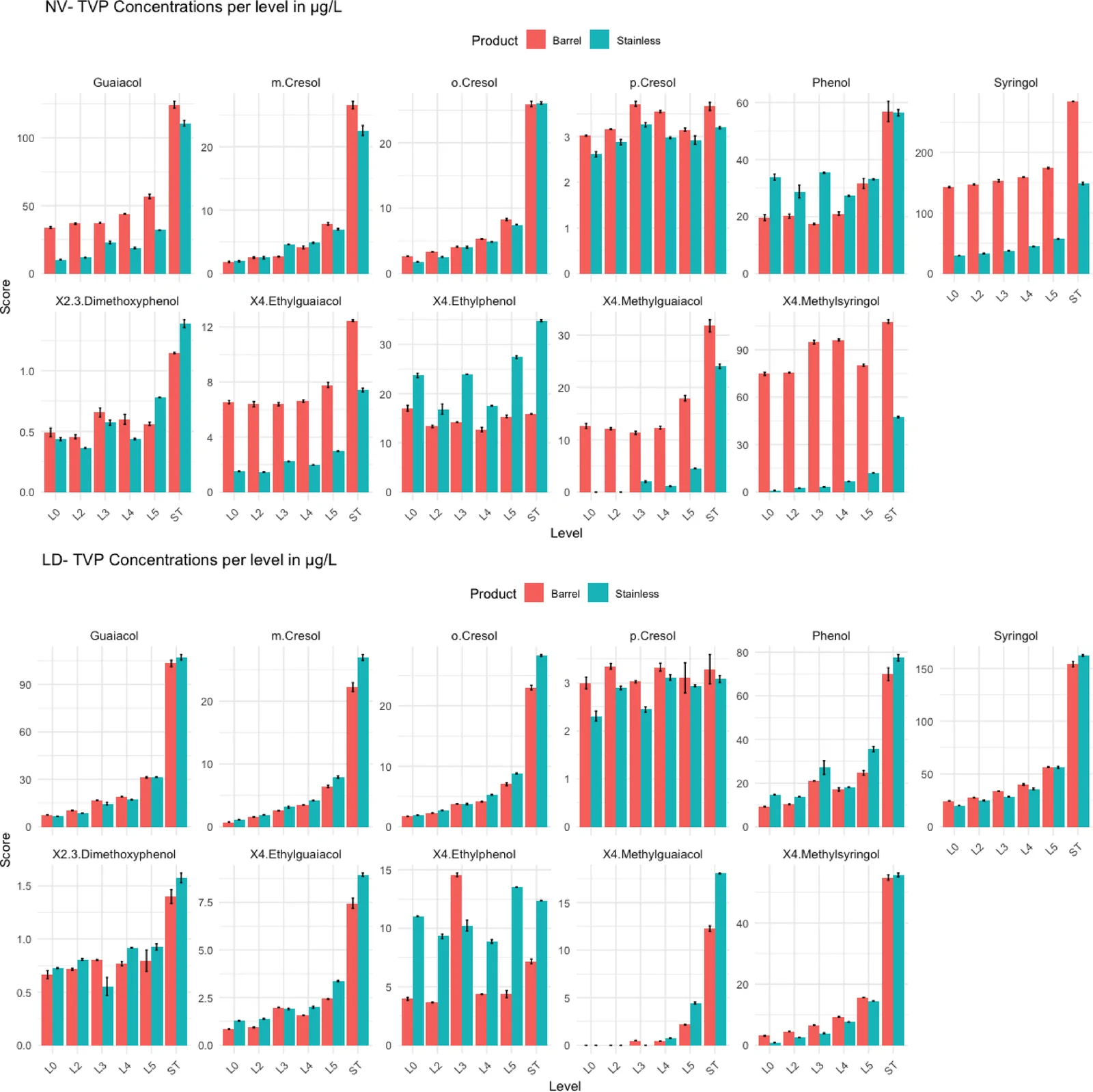
Total volatile phenol concentrations (μg/L) per smoke taint level, with standard errors, comparing the barrel-aged and stainless steel wines for the Napa Cabernet Sauvignon (NV, top) and Lodi Cabernet Sauvignon (LD, bottom).

There was an increase in concentration of free VPs with the increase of the amount of ST wine in the blend (Table 3). *p*-cresol had higher concentrations in the barrel treatment for both the Napa and the Lodi Cabernet Sauvignon; and in the Napa Cabernet Sauvignon, guaiacol, phenol, syringol, ethylguaiacol, methyguaiacol, and methylsyringol all had higher concentrations in the barrel-aged wines compared to the stainless-steel wines (Table 4). This is better illustrated in Fig. 1. Among the total VPs (Table 4), *p*-cresol had higher concentrations in the barrel treatment, while there was a higher concentration of ethylphenol in the stainless-steel wines for both the Napa and the Lodi Cabernet Sauvignon. In the Napa Cabernet Sauvignon wine, guaiacol, syringol, ethylguaiacol, methyguaiacol, and methylsyringol all had higher concentrations in the barrel-aged wines compared to the stainless-steel wines (Fig. 2). Guaiacol, ethylguaiacol, methylguaiacol, *p*-cresol and syringol are compounds known to be present in toasted barrels, which causes increases in their concentrations^41,60^. The observed differences in the free and total VPs comparing barrel and stainless-steel treatments were therefore expected.

**Table 3:** Mean free volatile phenol concentrations (μg/L) for all wines tested. Fisher’s LSD was used to determine differences between treatments (barrel aged or stainless steel) across the wines. L1 was not included in Fisher’s LSD calculation as it was not part of the descriptive analysis.

Wine	Guaiacol	4-Methylguaiacol	*o*-Cresol	Phenol	4-Ethylguaiacol	*p*-Cresol	*m*-Cresol	2.3-Dimethoxyphenol	4-Ethylphenol	Syringol	4-Methylsyringol
NV, Stainless steel											
NV_SS_L0	1.559 e	0.187 e	0.491 e	2.987 e	0.206 d	0.384 e	0.472 e	0.090 bc	1.080 e	17.842 e	1.108 d
NV_SS_L1	3.567 *	0.768 *	0.891 *	3.491 *	0.201 *	0.457 *	1.073 *	0.021 *	1.223 *	25.348 *	0.597 *
NV_SS_L2	4.371 de	1.275 de	1.204 de	3.961 de	0.244 d	0.426 de	1.469 de	0.018 c	1.278 de	19.998 de	0.822 d
NV_SS_L3	7.203 d	2.462 d	1.869 d	4.531 d	0.406 d	0.476 cd	2.477 d	0.040 bc	1.697 cd	23.318 d	1.789 cd
NV_SS_L4	13.484 c	5.084 c	3.425 c	6.386 c	0.747 c	0.544 c	4.654 c	0.075 bc	1.984 c	31.448 c	3.710 c
NV_SS_L5	23.846 b	9.414 b	6.044 b	9.135 b	1.319 b	0.641 b	8.425 b	0.138 b	3.035 b	42.141 b	6.858 b
NV_SS_ST	88.565 a	36.673 a	22.828 a	27.611 a	4.915 a	1.329 a	30.948 a	0.604 a	6.276 a	115.548 a	29.196 a
NV, Barrel aged											
NV_BL_L0	32.740 e	31.391 e	1.267 e	8.401 e	4.351 e	1.382 c	1.276 e	0.059 c	0.497 e	117.235 e	62.981 c
NV_BL_L1	35.400 *	33.247 *	1.736 *	9.023 *	4.632 *	1.475 *	1.937 *	0.119 *	0.624 *	128.825 *	80.050 *
NV_BL_L2	35.762 d	32.816 d	2.049 d	9.258 de	4.597 d	1.435 c	2.229 d	0.073 c	0.728 d	123.441 d	65.434 c
NV_BL_L3	36.380 d	33.584 d	2.086 d	9.561 d	4.677 d	1.416 c	2.236 d	0.085 c	0.742 d	125.251 d	71.751 b
NV_BL_L4	44.425 c	36.863 c	4.067 c	12.272 c	5.197 c	1.567 b	5.369 c	0.192 b	1.351 c	138.917 c	84.022 a
NV_BL_L5	52.421 b	39.675 b	6.392 b	13.432 b	5.642 b	1.601 b	8.478 b	0.203 b	2.062 b	144.067 b	67.936 bc
NV_BL_ST	110.776 a	63.027 a	21.501 a	28.962 a	9.490 a	2.258 a	30.156 a	0.719 a	6.300 a	227.336 a	84.631 a
LD, Stainless steel											
LD_SS_L0	1.124 f.	0.119 f.	0.873 e	1.911 f.	0.142 f.	0.205 d	0.363 f.	0.064 e	0.615 f.	10.138 f.	0.000 f.
LD_SS_L1	3.123 *	1.031 *	1.378 *	3.244 *	0.264 *	0.237 *	1.143 *	0.096 *	0.940 *	20.134 *	0.445 *
LD_SS_L2	3.849 e	1.689 e	1.618 de	4.223 e	0.334 e	0.186 d	1.587 e	0.128 d	0.826 e	13.890 e	1.002 e
LD_SS_L3	6.989 d	3.345 d	2.456 d	6.188 d	0.544 d	0.230 cd	2.783 d	0.119 d	1.141 d	16.540 d	2.075 d
LD_SS_L4	12.673 c	6.738 c	4.111 c	9.796 c	0.985 c	0.298 c	5.528 c	0.218 c	1.634 c	25.186 c	4.359 c
LD_SS_L5	24.664 b	12.996 b	7.053 b	18.062 b	1.809 b	0.466 b	10.188 b	0.286 b	2.328 b	39.588 b	9.934 b
LD_SS_ST	96.668 a	53.465 a	28.817 a	62.674 a	7.036 a	1.166 a	41.896 a	0.940 a	7.566 a	136.738 a	42.139 a
LD, Barrel aged											
LD_BL_L0	3.382 f.	1.595 f.	0.831 f.	2.484 e	0.221 f.	0.465 d	0.472 f.	0.073 de	0.523 f.	13.039 f.	1.536 f.
LD_BL_L1	5.137 *	2.392 *	1.183 *	4.745 *	0.324 *	0.504 *	1.112 *	0.029 *	0.514 *	20.505 *	2.224 *
LD_BL_L2	6.009 e	3.037 e	1.468 e	5.796 d	0.401 e	0.434 d	1.508 e	0.087 d	0.700 e	15.541 e	2.598 e
LD_BL_L3	8.957 d	4.672 d	2.367 d	6.377 d	0.606 d	0.520 c	2.723 d	0.066 e	0.895 d	19.629 d	3.979 d
LD_BL_L4	13.623 c	7.338 c	3.496 c	9.993 c	0.931 c	0.563 c	4.687 c	0.163 c	1.223 c	25.850 c	5.846 c
LD_BL_L5	24.093 b	13.000 b	6.152 b	18.085 b	1.636 b	0.710 b	8.991 b	0.264 b	2.006 b	39.788 b	10.386 b
LD_BL_ST	92.020 a	50.119 a	23.635 a	59.479 a	6.322 a	1.538 a	36.795 a	0.915 a	6.823 a	131.697 a	41.190 a

Within a column, means sharing superscripts are not significantly different (p > 0.05).

**Table 4:** Mean acid labile (total) volatile phenol concentrations (μg/L) for all wines tested. Fisher’s LSD was used to determine differences between treatments (barrel-aged or stainless steel) across the wines. L1 was not included in Fisher’s LSD calculation as it was not part of the descriptive analysis.

Wine	Guaiacol	4-Methylguaiacol	*o*-Cresol	Phenol	4-Ethylguaiacol	*p*-Cresol	*m*-Cresol	2.3-Dimethoxyphenol	4-Ethylphenol	Syringol	4-Methylsyringol
NV, Stainless steel											
NV_SS_L0	10.260 e	0.000 e	1.788 f.	33.843 b	1.507 e	2.613 c	1.939 d	0.436 d	23.687 c	29.450 f.	0.965 f.
NV_SS_L1	10.689 *	0.000 *	1.965 *	27.873 *	1.245 *	2.651 *	1.803 *	0.370 *	15.934 *	34.866 *	1.833 *
NV_SS_L2	11.730 e	0.000 e	2.555 e	28.791 c	1.458 e	2.878 b	2.494 d	0.363 e	16.858 d	33.133 e	2.459 e
NV_SS_L3	22.968 c	1.997 c	4.016 d	35.339 b	2.224 c	3.260 a	4.597 c	0.572 c	23.955 c	37.456 d	3.321 d
NV_SS_L4	18.772 d	1.112 d	4.835 c	27.252 c	1.963 d	2.974 b	4.859 c	0.437 d	17.540 d	44.581 c	6.620 c
NV_SS_L5	32.025 b	4.509 b	7.491 b	33.081 b	2.976 b	2.925 b	6.993 b	0.781 b	27.420 b	57.343 b	12.145 b
NV_SS_ST	110.761 a	24.048 a	26.080 a	56.427 a	7.420 a	3.197 a	22.564 a	1.389 a	34.821 a	148.916 a	47.417 a
NV, Barrel aged											
NV_BL_L0	33.998 d	12.631 c	2.670 f.	19.646 c	6.533 c	3.024 c	1.837 d	0.491 de	17.015 a	142.776 f.	74.778 d
NV_BL_L1	33.162 *	9.863 *	2.802 *	15.662 *	5.940 *	3.874 *	1.815 *	0.536 *	11.731 *	149.862 *	90.480 *
NV_BL_L2	36.819 d	12.087 c	3.341 e	20.143 c	6.386 c	3.164 c	2.523 d	0.456 e	13.340 cd	147.051 e	75.577 d
NV_BL_L3	37.324 d	11.319 c	4.102 d	17.424 c	6.390 c	3.720 a	2.671 d	0.656 b	14.201 c	153.053 d	94.806 b
NV_BL_L4	43.930 c	12.314 c	5.314 c	21.035 c	6.605 c	3.546 b	4.104 c	0.599 bc	12.692 d	159.125 c	96.216 b
NV_BL_L5	56.826 b	17.953 b	8.285 b	31.592 b	7.783 b	3.154 c	7.835 b	0.562 cd	15.382 b	174.337 b	80.243 c
NV_BL_ST	124.404 a	31.789 a	25.945 a	56.877 a	12.464 a	3.662 ab	26.581 a	1.147 a	15.902 b	284.328 a	107.882 a
LD, Stainless steel											
LD_SS_L0	6.543 e	0.000 d	1.919 f.	14.682 d	1.283 d	2.308 c	1.132 f.	0.726 c	11.035 c	20.185 f.	0.904 f.
LD_SS_L1	8.136 *	0.000 *	2.532 *	14.594 *	1.292 *	3.097 *	1.552 *	0.744 *	10.344 *	29.851 *	1.839 *
LD_SS_L2	8.662 e	0.000 d	2.731 e	13.721 d	1.385 d	2.896 b	1.908 e	0.807 bc	9.314 e	24.874 e	2.511 e
LD_SS_L3	14.625 d	0.000 d	3.753 d	27.172 c	1.910 c	2.445 c	3.105 d	0.553 d	10.232 d	28.356 d	3.919 d
LD_SS_L4	17.127 c	0.751 c	5.288 c	18.162 d	1.993 c	3.112 a	4.168 c	0.918 b	8.880 e	35.906 c	7.594 c
LD_SS_L5	31.439 b	4.433 b	8.827 b	35.584 b	3.369 b	2.940 ab	7.912 b	0.924 b	13.521 a	56.201 b	14.493 b
LD_SS_ST	107.224 a	18.063 a	28.325 a	77.434 a	8.952 a	3.081 ab	26.931 a	1.575 a	12.379 b	162.394 a	55.683 a
LD, Barrel aged											
LD_BL_L0	7.587 e	0.000 d	1.713 e	9.262 d	0.854 e	2.998 a	0.751 e	0.664 b	3.966 cd	24.526 e	3.087 f.
LD_BL_L1	12.103 *	0.000 *	2.416 *	16.346 *	1.502 *	3.095 *	1.535 *	0.706 *	10.558 *	34.853 *	4.211 *
LD_BL_L2	10.382 d	0.000 d	2.288 d	10.287 d	0.939 e	3.346 a	1.563 e	0.715 b	3.661 d	27.667 e	4.539 e
LD_BL_L3	16.790 c	0.494 c	3.795 c	21.086 bc	1.988 c	3.023 a	2.549 d	0.803 b	14.577 a	33.705 d	6.605 d
LD_BL_L4	18.984 c	0.428 c	4.151 c	17.068 c	1.574 d	3.328 a	3.489 c	0.768 b	4.367 c	40.085 c	9.292 c
LD_BL_L5	31.229 b	2.163 b	7.040 b	24.660 b	2.427 b	3.104 a	6.406 b	0.795 b	4.362 c	56.596 b	15.652 b
LD_BL_ST	103.302 a	12.251 a	23.003 a	69.856 a	7.455 a	3.286 a	22.204 a	1.400 a	7.170 b	154.101 a	54.856 a

Within a column, means sharing superscripts are not significantly different (p > 0.05).

For the volatile phenol glycosides (VPG) (Table 5), there was an increase in the concentrations of guaiacol glucoside, methylguaiacol rutinoside, syringol gentiobioside, and methylsyringol gentiobioside with the increase in the amount of ST wine present in the blend. When comparing the treatment between barrel-aged and stainless-steel wines, guaiacol rutinoside and phenol rutinoside had higher concentrations in the barrel treatments as compared to the stainless-steel treatments in both the Napa Cabernet Sauvignon and Lodi Cabernet Sauvignon, though these levels remained relatively similar across dilutions.

**Table 5:** Mean volatile phenol glycoside concentrations (μg/L) for all wines tested. Fisher’s LSD was used to determine differences between treatments (barrel-aged or stainless steel) across the wines. L1 was not included in Fisher’s LSD calculation as it was not part of the descriptive analysis.

Wine	Guaiacol gentiobioside	Syringol gentiobioside	Guaiacol glucoside	Phenol rutinoside	Guaiacol rutinoside	4-Methylsyringol gentiobioside	Cresol rutinoside	4-Methylguaiacol rutinoside
NV, Stainless steel								
NV_SS_L0	0.000 d	2.685 d	0.762 e	1.431 c	1.793 d	0.143 e	15.606 c	0.868 c
NV_SS_L1	0.000 *	2.964 *	1.250 *	1.295 *	1.232 *	0.144 *	19.203 *	0.991 *
NV_SS_L2	0.000 d	2.955 c	1.298 d	1.500 c	2.117 bc	0.158 e	17.249 c	0.822 c
NV_SS_L3	0.031 c	2.987 c	1.480 d	1.348 c	2.094 c	0.206 d	20.283 b	0.833 c
NV_SS_L4	0.052 c	3.131 c	2.055 c	1.513 c	1.657 d	0.277 c	17.722 c	0.900 c
NV_SS_L5	0.126 b	3.592 b	3.188 b	1.810 b	2.262 ab	0.352 b	21.841 b	1.152 b
NV_SS_ST	0.500 a	6.528 a	10.996 a	2.204 a	2.348 a	1.015 a	27.644 a	1.856 a
NV, Barrel aged								
NV_BL_L0	0.000 c	2.783 d	1.144 e	1.660 b	2.142 b	0.174 e	16.587 b	0.570 c
NV_BL_L1	0.000 *	2.522 *	1.245 *	1.708 *	1.367 *	0.155 *	14.922 *	0.920 *
NV_BL_L2	0.000 c	2.757 d	1.556 d	1.747 b	2.191 b	0.206 de	17.746 b	0.908 b
NV_BL_L3	0.000 c	2.879 cd	1.493 d	1.655 b	2.279 b	0.226 d	13.578 cd	0.834 b
NV_BL_L4	0.064 b	3.098 c	2.569 c	1.410 c	2.196 b	0.288 c	12.650 d	0.925 b
NV_BL_L5	0.064 b	3.456 b	3.546 b	1.615 b	2.573 a	0.350 b	14.250 c	0.986 b
NV_BL_ST	0.506 a	6.282 a	11.344 a	2.135 a	2.620 a	1.022 a	19.517 a	1.895 a
LD, Stainless steel								
LD_SS_L0	0.000 e	4.254 cd	0.532 e	1.730 a	1.932 b	0.264 e	11.737 cd	0.796 d
LD_SS_L1	0.051 *	4.254 *	0.946 *	1.920 *	1.435 *	0.334 *	11.696 *	1.061 *
LD_SS_L2	0.024 d	4.057 d	0.973 d	1.846 a	2.026 b	0.277 de	11.458 d	1.001 c
LD_SS_L3	0.062 c	4.463 bc	1.220 d	1.899 a	1.818 b	0.328 d	12.655 c	1.032 c
LD_SS_L4	0.008 de	4.267 cd	1.996 c	1.718 a	1.811 b	0.391 c	11.891 cd	1.010 c
LD_SS_L5	0.093 b	4.693 b	3.058 b	1.737 a	1.825 b	0.459 b	17.421 a	1.288 b
LD_SS_ST	0.372 a	7.678 a	10.345 a	1.766 a	2.343 a	1.236 a	14.211 b	2.429 a
LD, Barrel aged								
LD_BL_L0	0.000 c	4.120 cd	1.057 e	2.062 c	2.333 a	0.278 d	12.117 bc	0.870 d
LD_BL_L1	0.000 *	3.919 *	1.162 *	1.601 *	1.676 *	0.214 *	10.669 *	0.860 *
LD_BL_L2	0.000 c	4.066 d	1.173 e	2.174 bc	2.391 a	0.284 d	12.741 bc	0.919 cd
LD_BL_L3	0.000 c	3.966 d	1.468 d	2.069 c	2.183 a	0.315 d	14.681 a	0.923 cd
LD_BL_L4	0.000 c	4.330 c	1.863 c	2.398 a	2.298 a	0.375 c	11.141 c	1.021 c
LD_BL_L5	0.056 b	4.884 b	3.268 b	2.264 ab	2.305 a	0.499 b	13.853 ab	1.234 b
LD_BL_ST	0.324 a	7.254 a	10.057 a	2.094 c	2.211 a	1.163 a	11.770 c	2.439 a

Within a column, means sharing superscripts are not significantly different (*p* > 0.05).

### Sensory properties of the wines

**Fig. 3:**
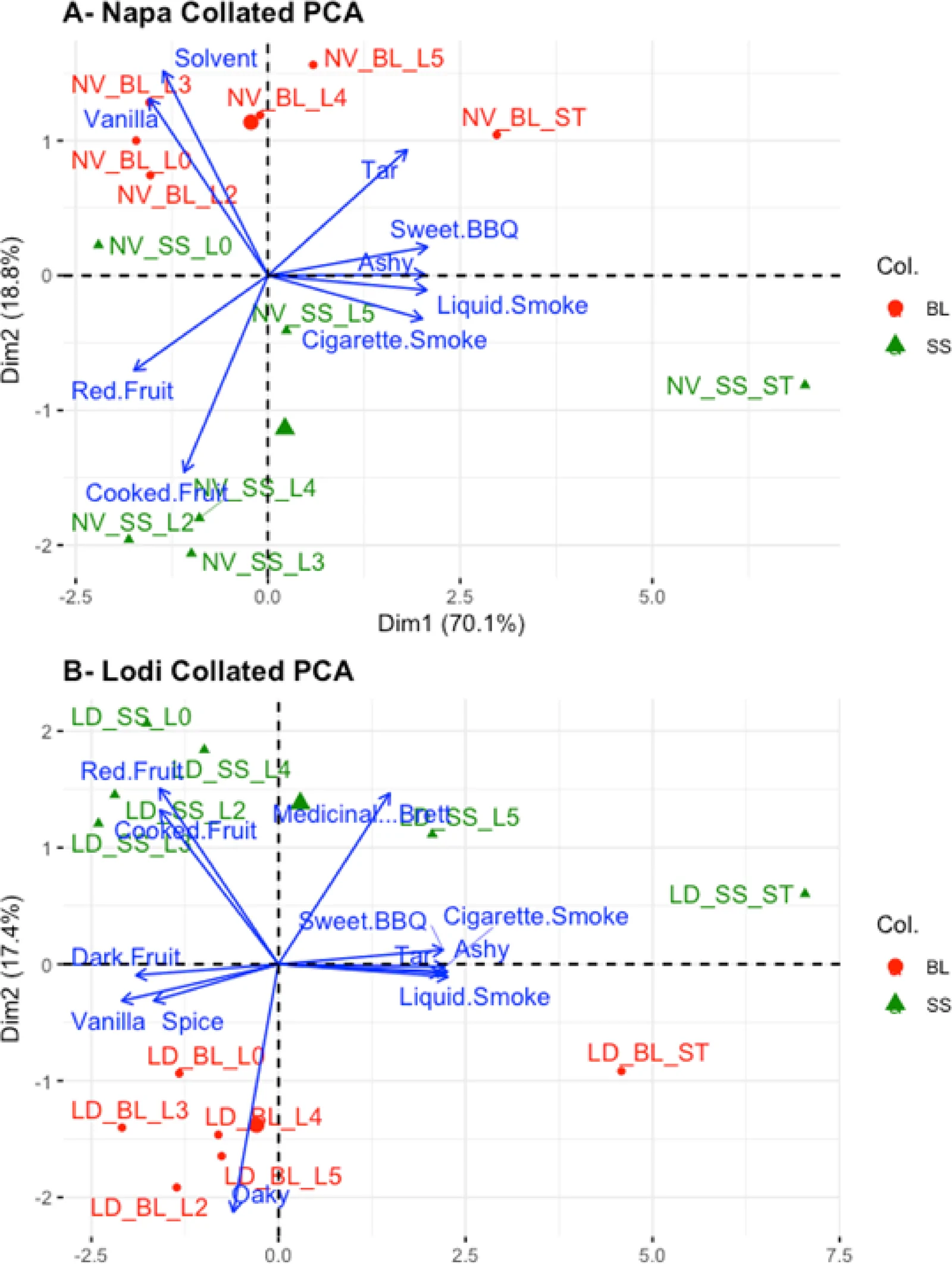
Principal component analysis biplot of the matrix of mean (significant) sensory attribute intensities across wines for the Napa Cabernet Sauvignon (**A**) and Lodi Cabernet Sauvignon (**B**) wines scaled to unit variance. Barrel-aged wines are shown in red and stainless-steel wines in green.

**Fig. 4:**
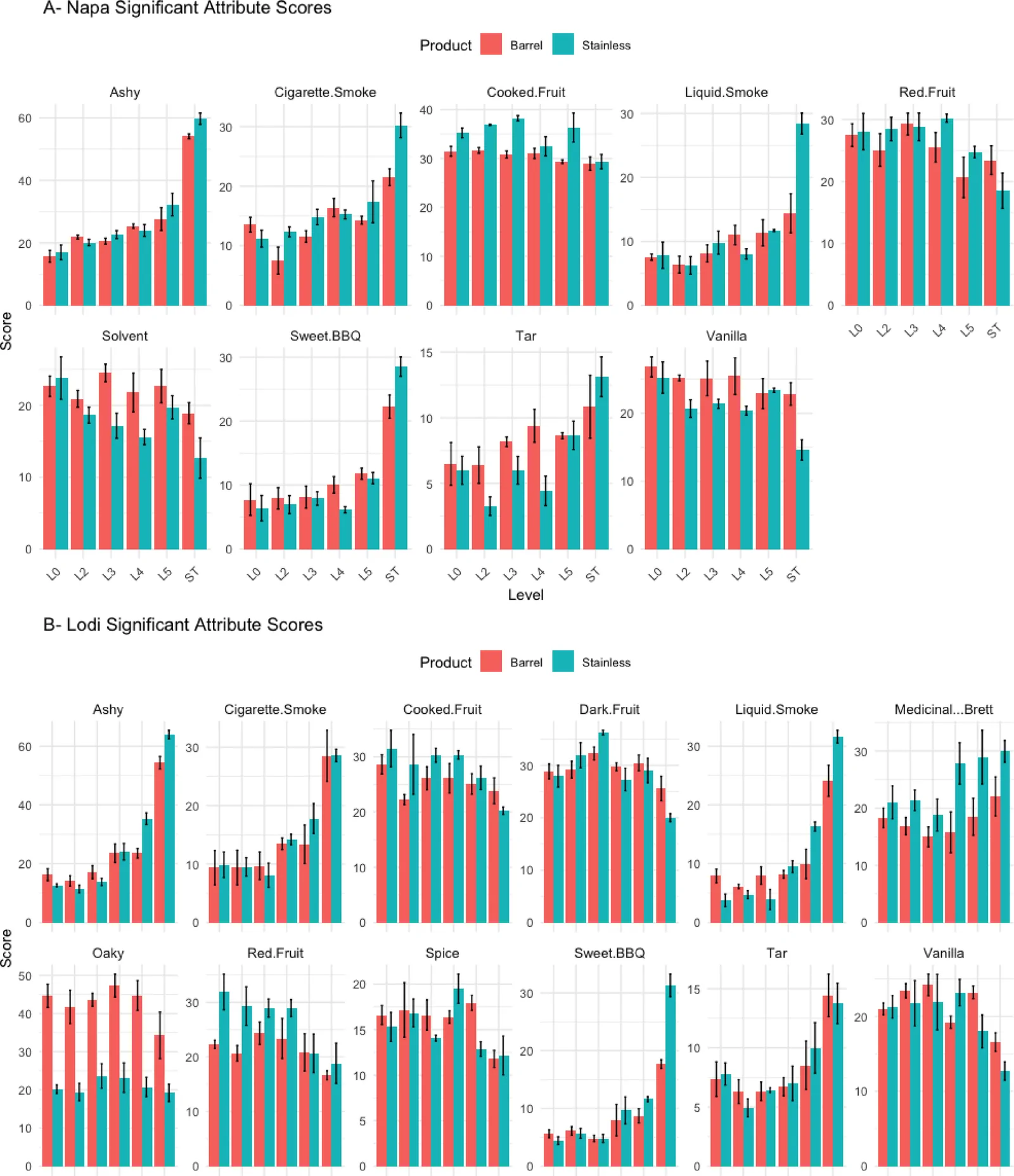
Napa Valley Cabernet Sauvignon (**A**) and Lodi Cabernet Sauvignon (**B**) wines mean intensity scores of significant sensory attributes with standard errors, comparing the barrel-aged (orange) and stainless steel (green) treatments.

A principal component analysis (PCA), scaled to unit variance, can be used to visualize the differences among the wines. For the full-bodied Napa Cabernet Sauvignon (Fig. 3A), PCs 1 and 2 accounted for 70.1% and 18.8% of the variance in the descriptive data, respectively, totaling 88.9%. Two main features of the PCA are worth highlighting. First, the wines were differentiated mainly across PC1, with one end associated with fruity and vanilla notes, and the other end with smoky (“sweet bbq”, “ashy”, “liquid smoke”, and “cigarette smoke”) attributes. Second, there was a separation between the oak barrel-aged wines and the stainless-steel wines along PC2. The barrel-aged wines had lower smoke-related mean attribute scores than the stainless-steel wines (Fig. 4A), for “ashy”, “cigarette smoke” and “liquid smoke”, indicating some level of oak mitigation of the smoke taint in the Cabernet Sauvignon wines we studied (Supplementary Table S3). From the PCA, it is evident that the main difference in smoke perception was observed in the ST wines. For the remaining wines, the smoke-related terms appear closer together, consistent with the smaller differences observed in their mean scores (Supplementary Table S4). The oak barrel-aged wines also had lower fruit attribute intensities, such as red fruit, compared to the stainless-steel wines as seen in Fig. 4A.

In the medium-bodied Lodi Cabernet Sauvignon (Fig. 3B), PCs 1 and 2 accounted for 68.5% and 17.4% of the variance, respectively, totaling 85.9%. Similar to the Napa wines, there was a separation between the oak barrel-aged wines and the stainless-steel wines along PC2, but the wines were differentiated mainly across PC1 as a function of smoke taint, with one end of PC1 associated with fruity and vanilla attributes, and the other with smoke-related flavors. The “medicinal/brett” character also appeared to be more associated with the stainless-steel wines, while the barrel treatments led to more oak flavors. This sensory character, “Medicinal/Brett” is higher in the stainless-steel aged Lodi wines compared to the barrel-aged wines (Fig. 4B). This is in alignment with the chemical analysis above where the concentration of 4-ethylphenol (Figs. 1 and 2), was lower in the barrel aged wines than the stainless-steel counterpart. The Lodi barrel-aged wines, in Fig. 4B, again had lower smoke-related mean attribute scores for “ashy”, “cigarette smoke”, “liquid smoke” and “sweet BBQ” than the stainless-steel wines (Supplementary Table S4). And again, from the PCA, it is evident that the main difference in smoke perception was seen with the ST wines.

In sum, the main differences among the wines were along smoke vs. fruity attributes. The aging in oak did contribute some notable characteristics as well, which appeared to lower the perceived intensities of smoke character, particularly the “ashy” character, in the barrel-aged wines. These higher smoke impacted barrel-aged wines were less smoky overall than their stainless-steel counterparts.

### Sensory quality of the wines

**Fig. 5:**
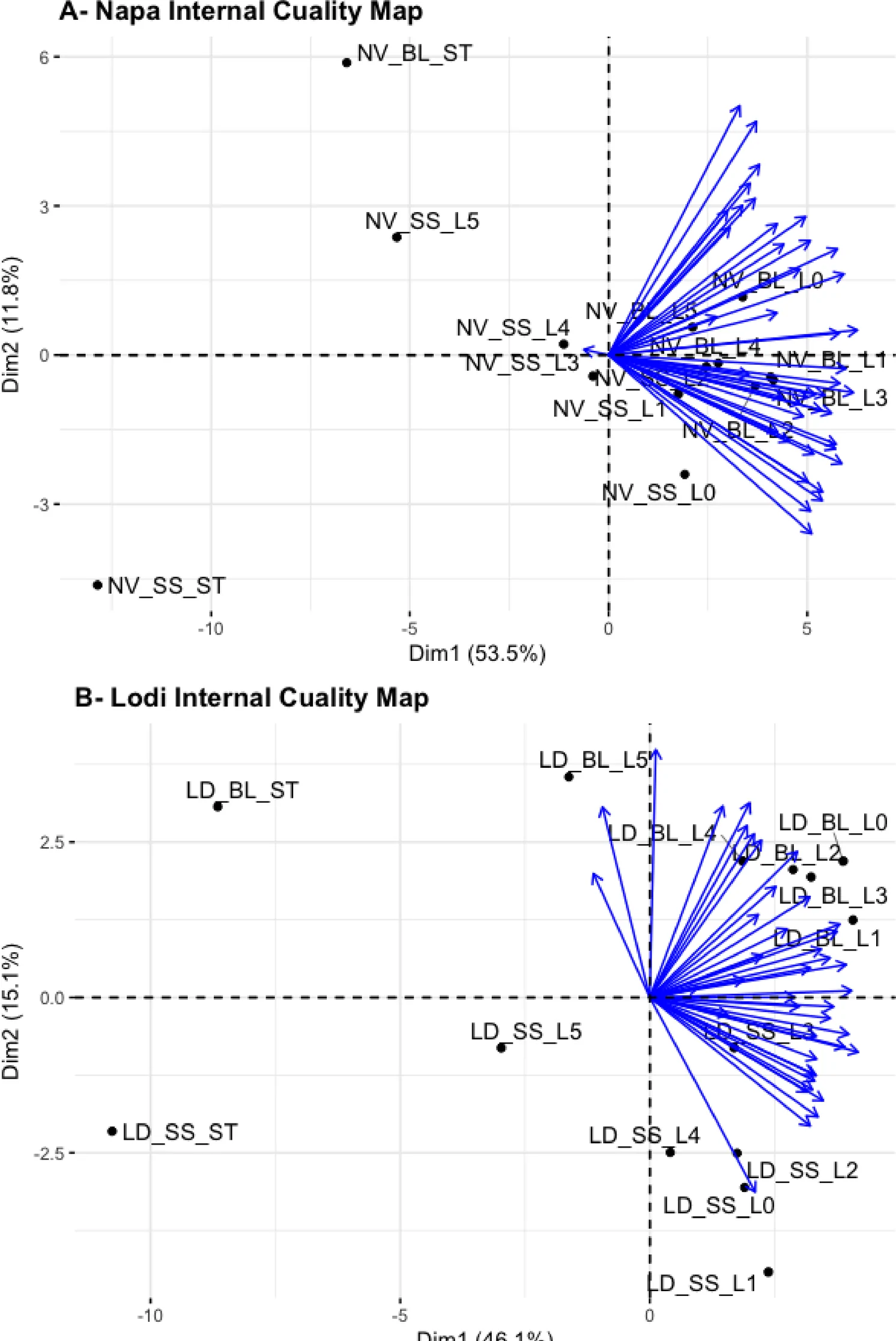
Internal Quality map of the Napa (**A**) and Lodi (**B**) wines, showing the experts (as quality vectors) and the barrel-aged and stainless-steel wines. The Internal Quality map is a PCA biplot of the matrix of expert quality ratings across the wines. The arrows represent loadings which are experts, and the dots represents objects which are the wines.

**Fig. 6:**
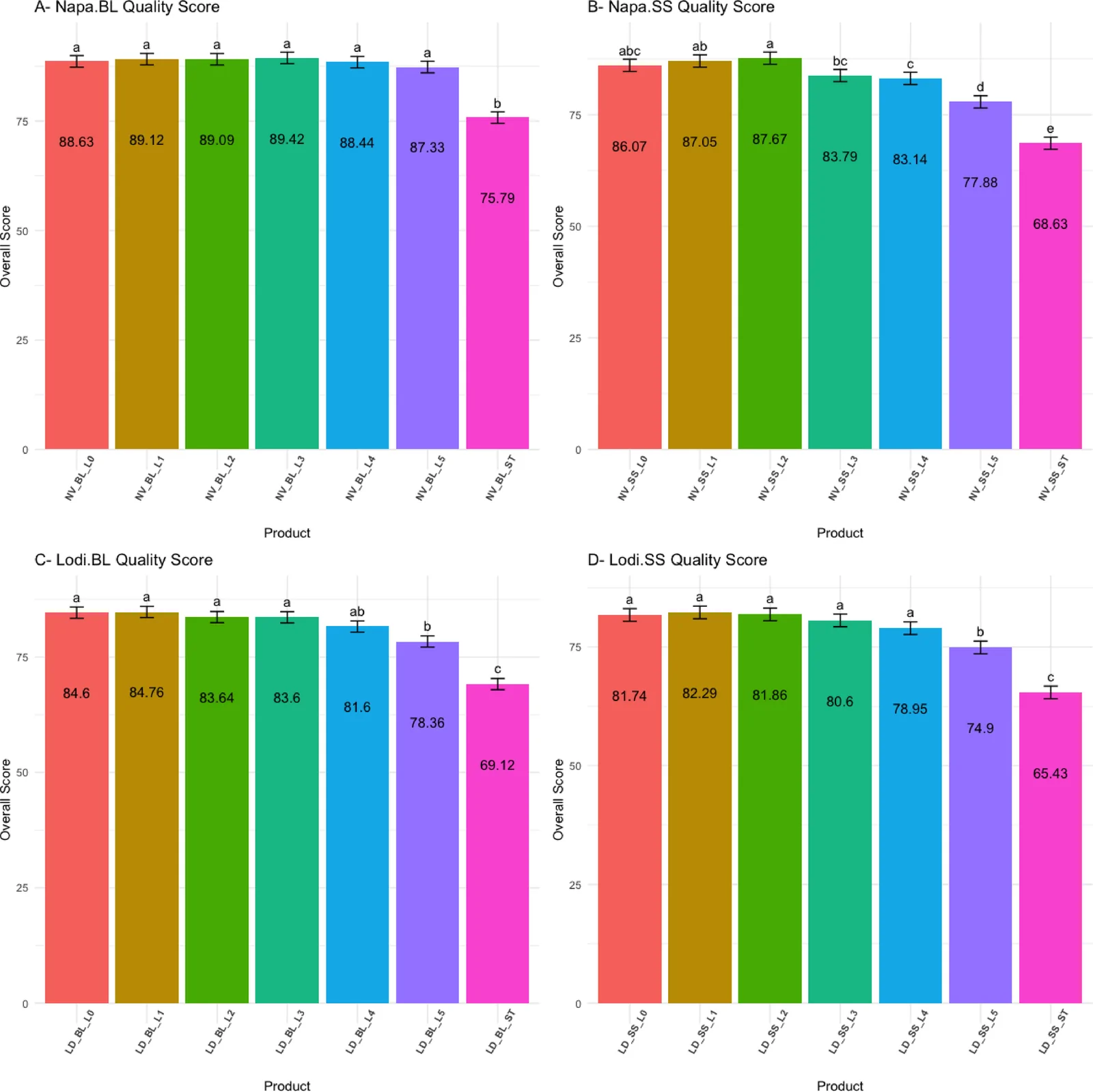
Mean quality ratings for Cabernet Sauvignon wines with varying levels of smoke taint – (**A**) Napa barrel-aged wines; (**B**) Napa stainless-steel wines; (**C**) Lodi barrel-aged wines; (**D**) Lodi stainless-steel wines.

The internal quality map (or PCA of the expert quality ratings across the wines) in Fig. 5 shows that the quality vectors representing each expert’s main direction of quality consistently point away from the smoke-tainted wines, regardless of whether those were from Napa or Lodi, or made in stainless steel or barrel-aged. There were some differences in the quality ratings based on the wine origin and treatment, however, as per the respective positions of the wines on the PCA biplot. Those differences are best illustrated in the bar graphs showing the mean quality ratings for the wines in Fig. 6A and B. In the Napa wines, wines with lower percentages of ST wines had higher quality scores as compared to the fully smoke-tainted (ST) wines or the L5 stainless-steel wine. For the barrel-aged wines, only the ST wine was rated significantly lower in quality compared to the other wines. For the stainless-steel wines, however, the first significant drop in quality was seen with wine L4, two Fisher’s LSD away from the other wines. Of note, we observed that the quality scores for stainless-steel L1 and L2 wines, and barrel-aged L1, L2, and L3 wines were higher than those of their respective L0 counterparts, suggesting that a very low level of smoke taint resulted in a higher perceived quality

For the Lodi wines, the internal quality map (Fig. 5B) is similar to that of the Napa wines (Fig. 5A) in that the wines with a higher percentage of smoke taint (L5 and ST) had lower quality scores, and the wines with lower smoke taint amounts were rated higher. ST and L5 Lodi wines were rated significantly lower in quality than the Napa wines in the set (Fig. 6). Again, L1 barrel-aged wine (Fig. 6C) and L1 and L2 stainless-steel wines (Fig. 6D) were rated higher than the control (L0) wines. The differences in quality ratings were lesser than for the Napa wines, which may have been due to the “thinner” wine matrix and lower overall scores to begin with.

**Fig. 7:**
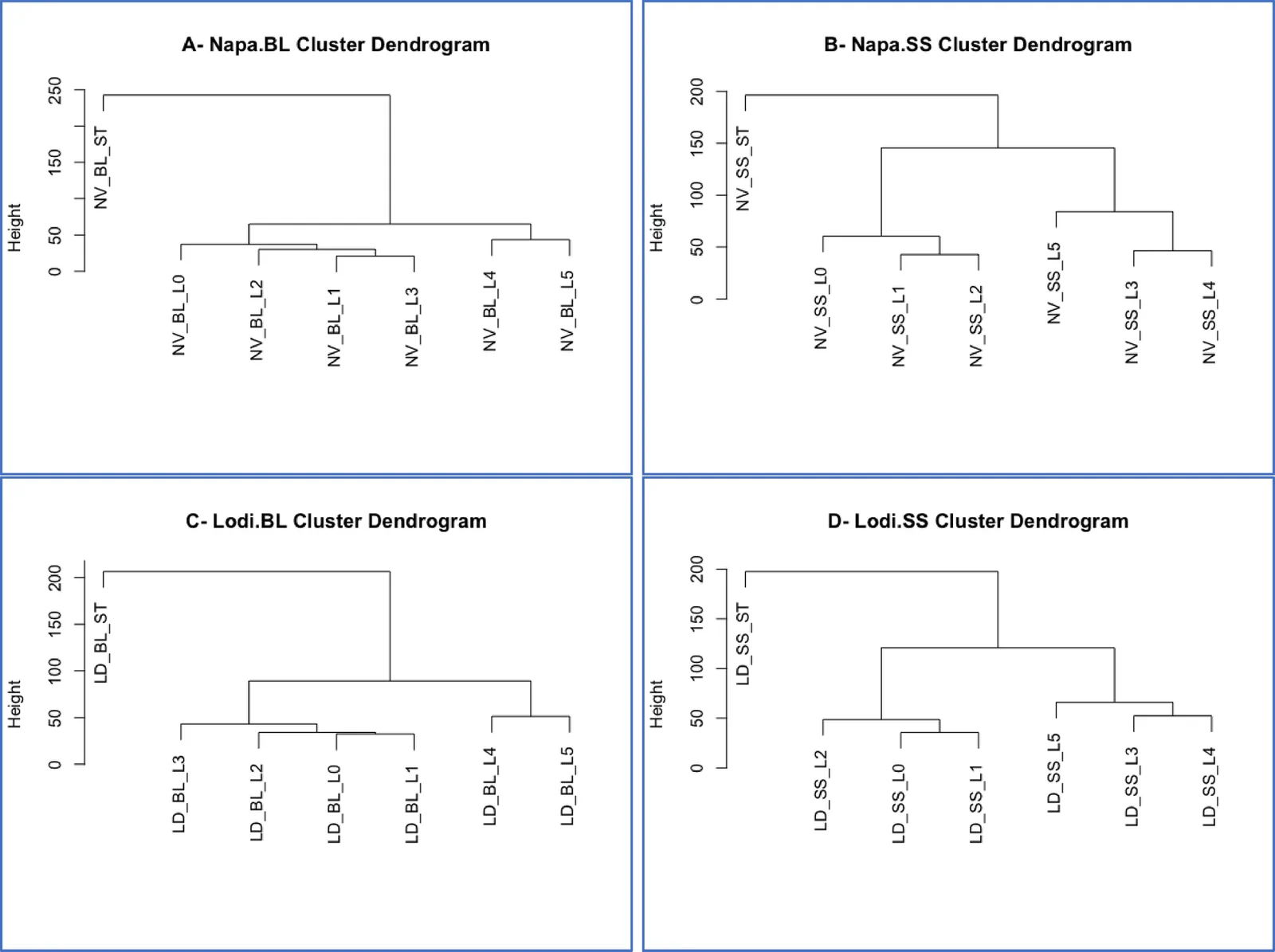
Quality ratings dendrograms for Cabernet Sauvignon wines with varying levels of smoke taint – (**A**) Napa barrel-aged wines; (**B**) Napa stainless-steel wines; (**C**) Lodi barrel-aged wines; (**D**) Lodi stainless-steel wines.

These wines were clustered based on their mean quality scores (Fig. 7), showing three clusters. The ST wine, being the most impacted wine, was clustered on its own across all treatments. In both the barrel-aged Napa (Fig. 7A) and Lodi (Fig. 7C) wines, the other two clusters are made up by first L0, L1, L2, and L3 wines forming one cluster, and the L4 and L5 wines forming another cluster. In contrast, in the stainless-steel Napa (Fig. 7B) and Lodi (Fig. 7D) wines, the other two clusters are made up by first L0, L1, and L2 wines forming one cluster, and the L3, L4, and L5 wines forming another cluster. This shows differences in the perception of quality between the barrel and stainless-steel wines at the various levels, regardless of the location from which the grapes came.

**Fig. 8:**
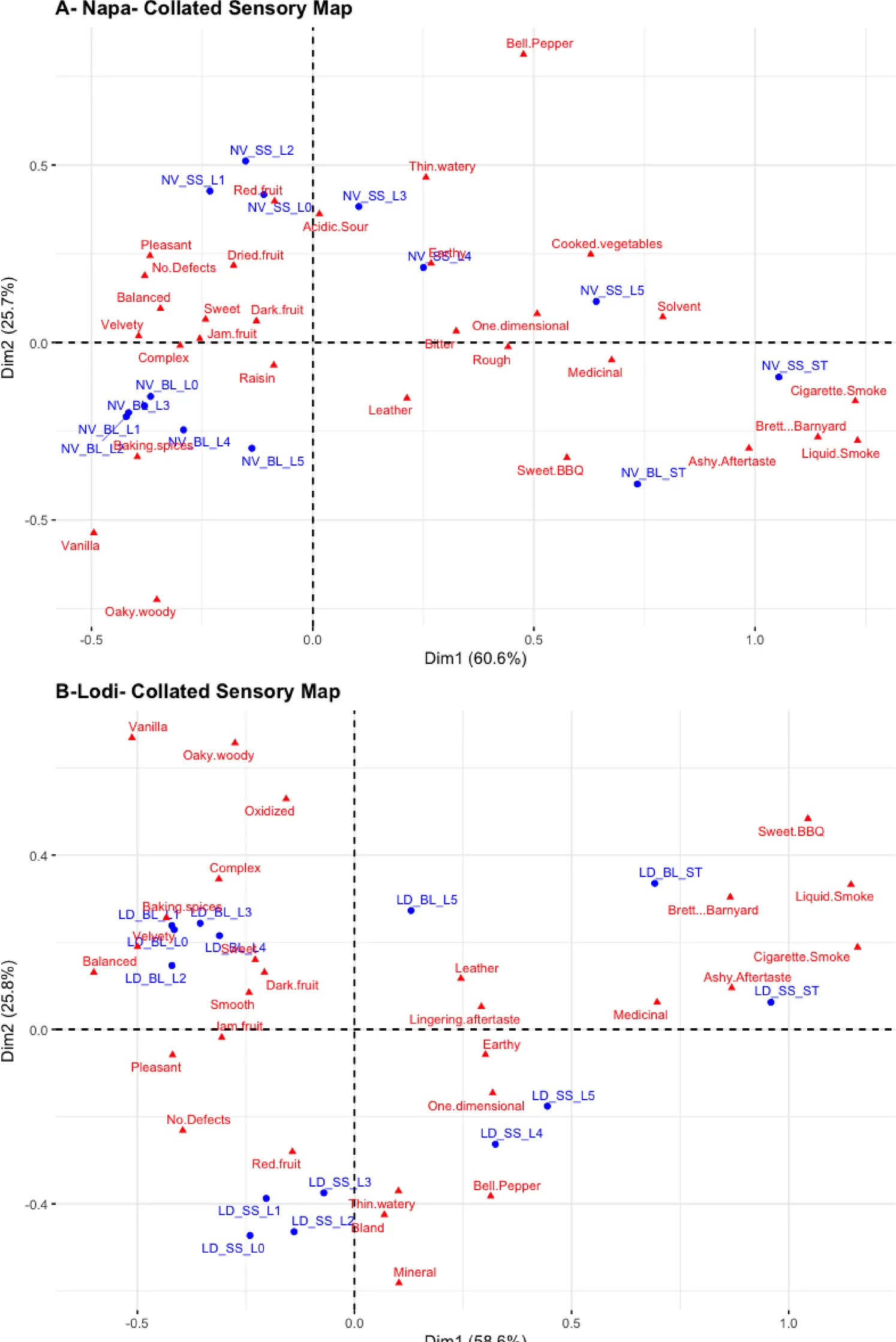
Sensory map derived from correspondence analysis of the experts’ CATA selections showing both the sensory and holistic attributes and the wines for (**A**) Napa wines, and (**B**) Lodi wines. The sensory terms are in red triangles representing the loadings and the wines in blue dots are the individuals.

In the sensory map for the Napa wines that we derived by correspondence analysis from the matrix of CATA selections by the experts (Fig. 8A), and similar to the PCA of the descriptive analysis ratings shown in Fig. 3A, there was a split between the SS and BL wines along Dimension 2. Dimension 1 contrasted fruity and smoke-related attributes. The barrel-aged wines with a low percentage of ST wine blended in were associated with “spice” and seen as “complex”. The stainless-steel wines were described as “red fruity” and “acidic”. For the Lodi wines (Fig. 8B), dimension 1 separated the wines along increasing smoke concentration, and Dimension 2 separated them according to the treatment. Importantly, for both sets of wines, ST wines were described as “ashy”, “cigarette smoke”, “liquid BBQ”, and “sweet BBQ”, which are similar descriptors to those of the Napa Cabernet Sauvignon and are consistent with the DA outcomes.

### Relating sensory, compositional, and quality data

**Fig. 9:**
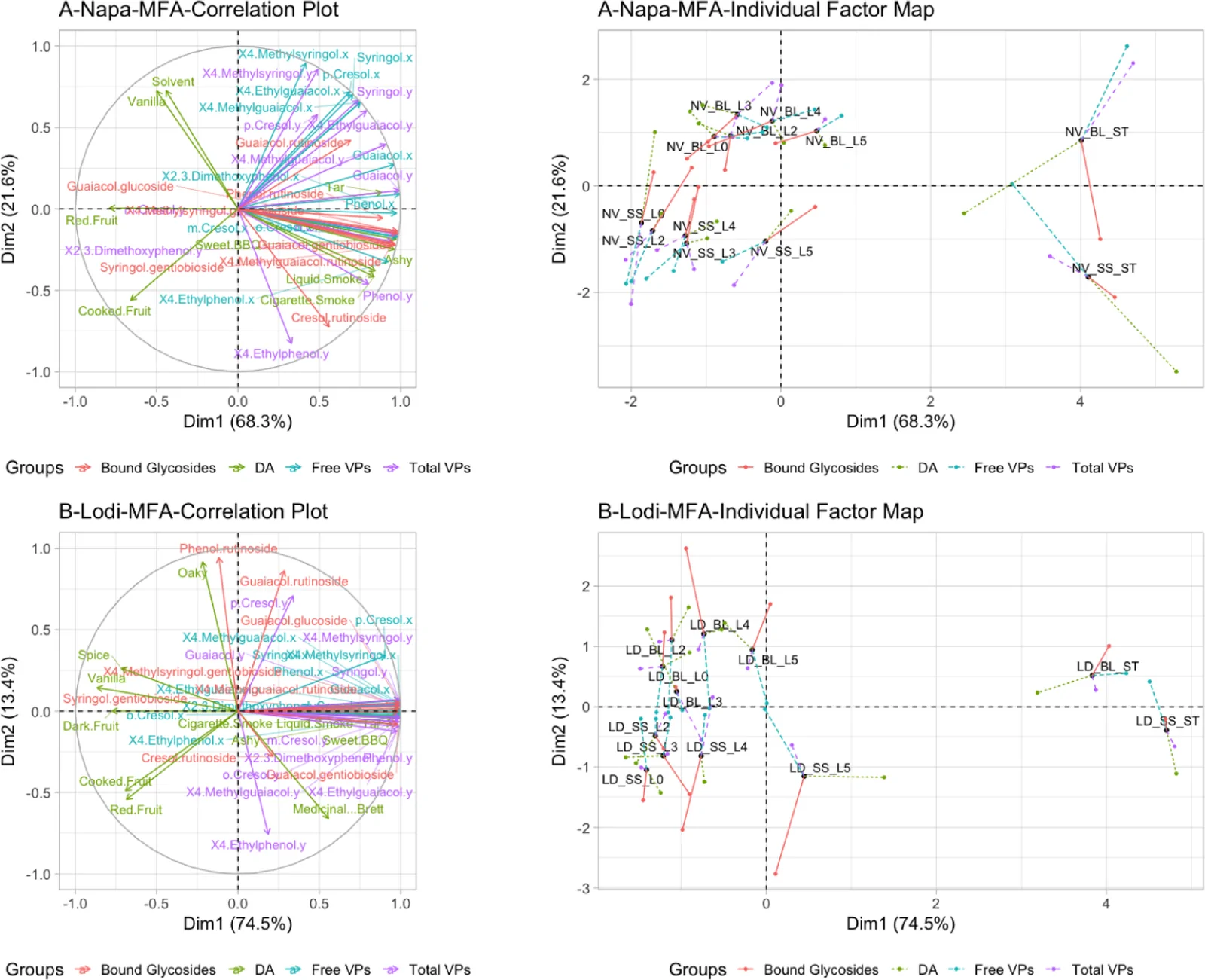
Multiple factor analysis of Napa Cabernet Sauvignon (**A**) and Lodi Cabernet Sauvignon (**B**) descriptive analysis and chemistry data, relating significant sensory attributes from the DA, individual bound glycosides, free VPs, and total VPs. Variables are shown as vectors on the left, and wines on the right are positioned according to the consensus from four datasets. Dotted lines indicate the distance between each dataset-specific position and the consensus.

Multiple factor analysis was first used to relate the descriptive analysis data to the compositional data across both Napa and Lodi wines. In the Napa wines (Fig. 9A), the MFA represents 68.3% (Dim1) and 21.6% (Dim2) for a total of 89.9% of the variance in the data explained, and in the Lodi wines (Fig. 9B), the MFA represents 74.5% (Dim1) and 13.4% (Dim2) for a total of 87.9% of the total variance in the data explained. In both plots, smoke attributes, “ashy”, “cigarette smoke”, “sweet BBQ”, and “tar” were well correlated with the free VPs, total VPs, and the glycosides. The wines on the positive side of the MFA’s first axis, NV_SS_ST, NV_BL_ST, LD_BL_ST, and LD_SS_ST for both Napa and Lodi wines were described as “ashy”, “cigarette smoke”, “sweet BBQ”, “tar”, and all had high levels of free, total, and bound glycosides. Interestingly, there is a close relation between p-cresol and guaiacol rutinoside in both wines, as well as among cresol rutinoside, 4-ethylphenol (both wines) and the “medicinal/Brett” character (Lodi wine). Conversely, it also confirms that the fruity and positive terms “dark fruit”, “red fruit”, “cooked fruit” and “vanilla” were not driven by any of the smoke chemical marker compounds.

**Fig. 10:**
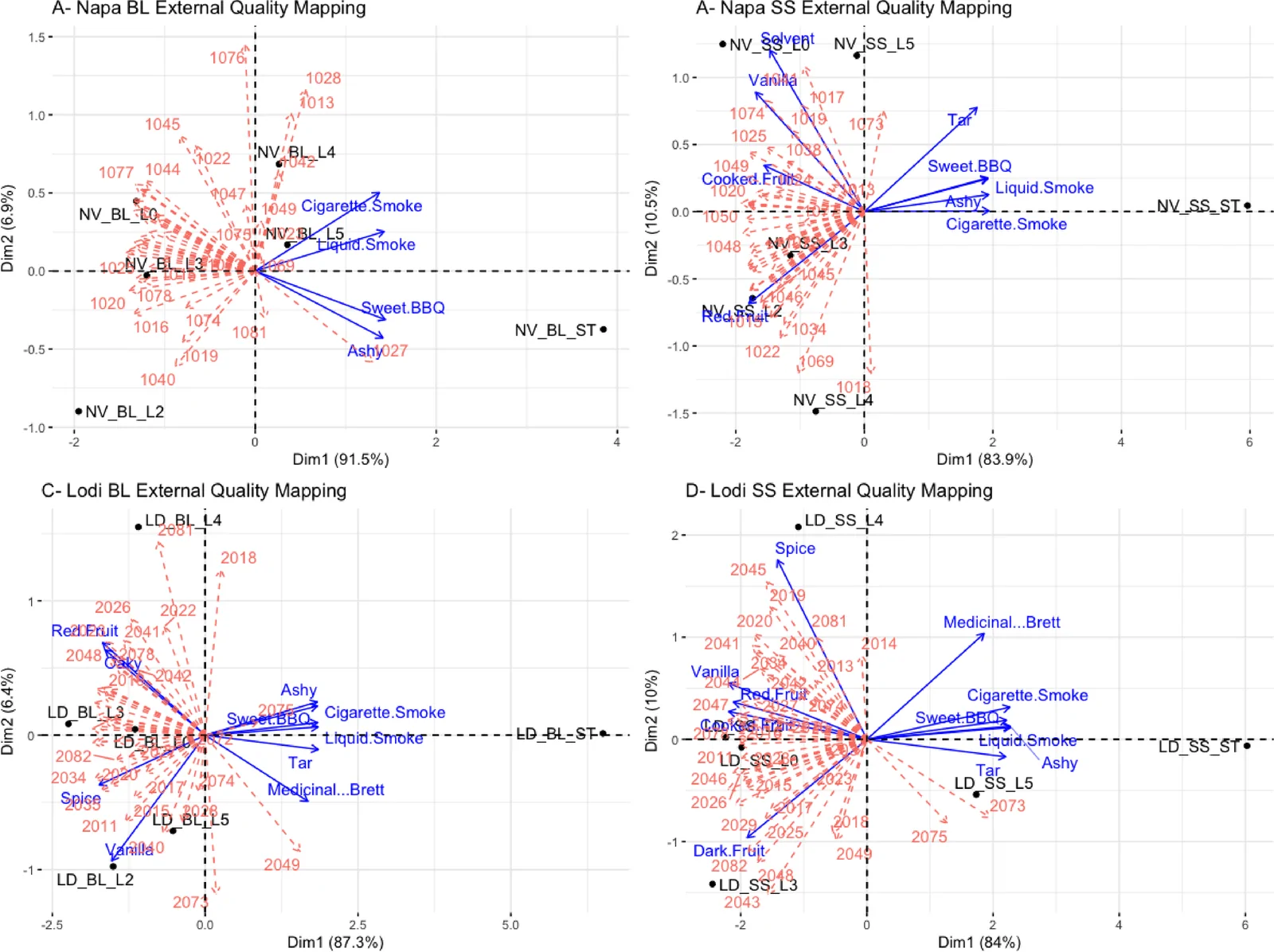
External quality map of the Napa Cabernet Sauvignon wines, barrel-aged (**A**) and stainless steel (**B**), and Lodi Cabernet Sauvignon, barrel-aged (**C**) and stainless steel (**D**). Individual PCAs from the barrel-aged or stainless steel descriptive analysis are shown, with the expert quality ratings regressed onto the first two PCs.

Second, the descriptive analysis and quality ratings on a 100-point scale were related using external quality mapping^47^. Here, we show two external quality maps, one for barrel-aged wines and the other for stainless-steel wines, as the context provided for the quality assessments was different. The barrel-aged wines were rated as finished wines that had just been released to the market, while the stainless-steel wines were represented as wines that were about to undergo barrel aging. In this case, they were rated for their promise in terms of their potential before aging. In the stainless-steel wines (Fig. 10), and for both Napa (Fig. 10B) and Lodi (Fig. 10D) wines, the wine experts were consistent in their ratings, as shown by the proximity of their quality vectors on the biplots. Most of their quality ratings gravitated towards the lower smoke-taint dilution wines, which had fewer smoke-related traits. In the barrel-aged Napa (Fig. 10A) and Lodi wines (Fig. 10C), there again was general agreement that the wines with lesser levels of smoke taint (L0, L2, L3) were consistently characterized as fruity rather than smoky.

**Fig. 11:**
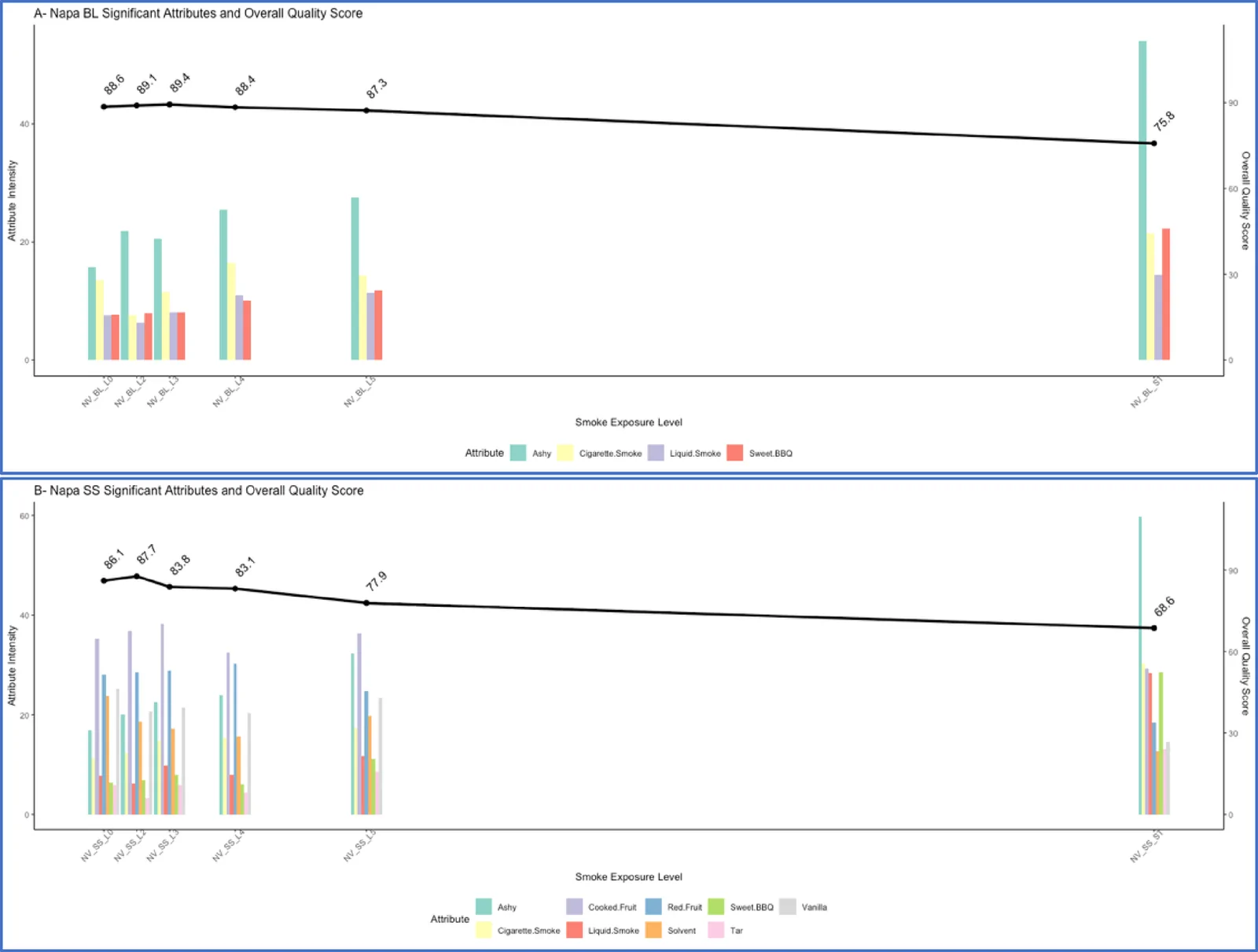

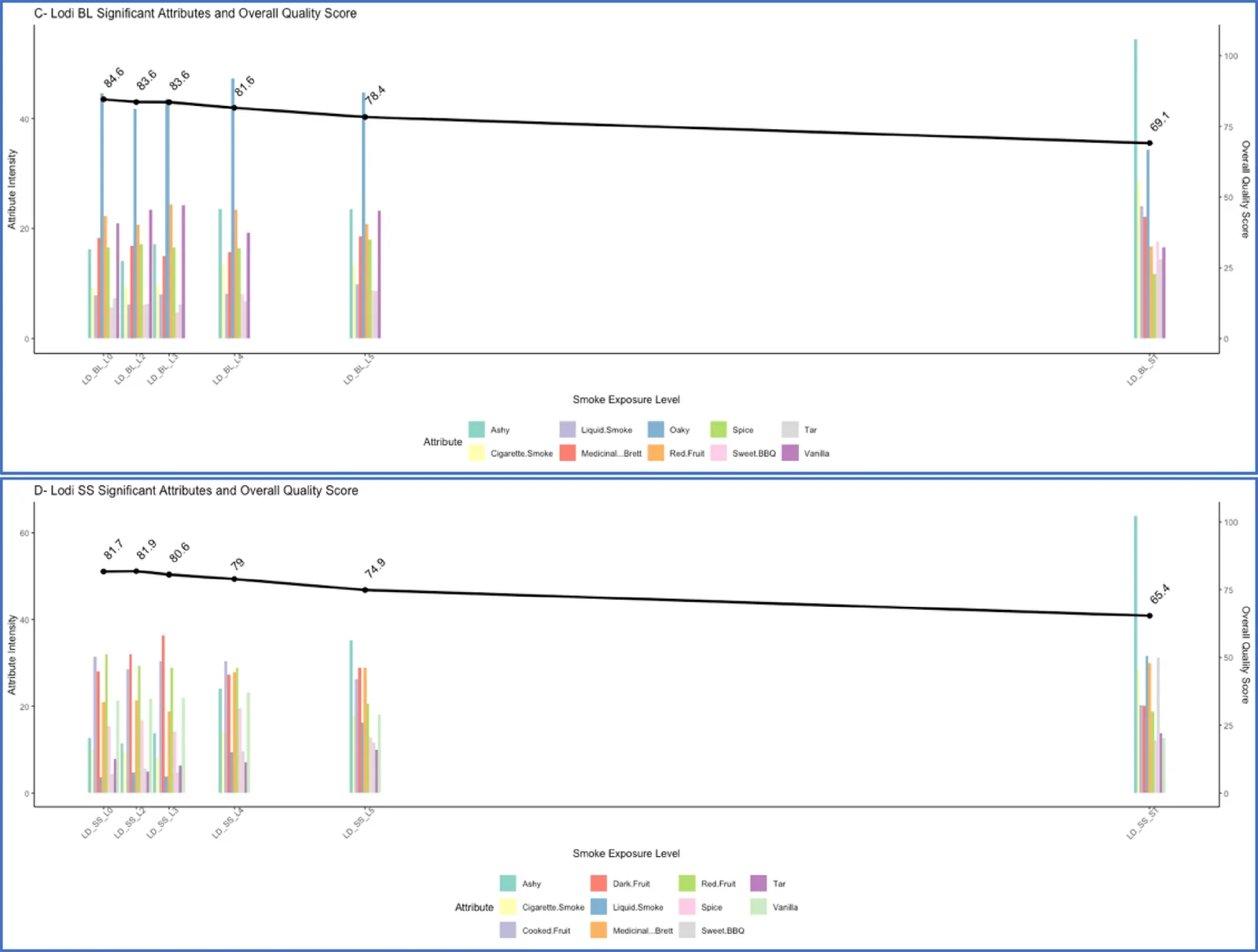
Mean quality ratings (top line) and mean sensory attribute intensities (bar graphs below) for the Napa Cabernet Sauvignon wines, (**A**) barrel-aged and (**B**) stainless steel; Lodi Cabernet Sauvignon wines, (**C**) barrel-aged and (**D**) stainless steel.

Third, to better visualize the differences among the wines, the mean overall sensory attribute intensities and a linearized scale of the mean quality rating are shown for each wine in Fig. 11. For the Napa wines, there was a negative relationship between the overall quality score and the increased percentage of smoke-impacted wine in the wine. As the amount of smoke-impacted wine increased, the ashy and smoke-related attributes increased in intensity, which was coupled with a decrease in the overall quality score. This was consistent for both the barrel-aged (Fig. 11A), and stainless-steel (Fig. 11B) treatments. At low dilution percentages, there was a slight increase in the overall quality score in NV_SS_L2, NV_BL_L2, and NV_BL_L3 over the base wine L0. In the stainless-steel wine, the first drop in quality score was recorded at L3 as compared to the barrel-aged wine, for which the quality score remained relatively similar for all dilutions up to NV_BL_L5.

In the Lodi wines, there also was a negative relationship between the overall quality score and the increased percentage of smoke-impacted wine in the mix. However, there was no increase in the quality score for the barrel-aged wine (Fig. 11C) at the low dilutions and an increase of 0.2 points for the stainless-steel wines (Fig. 11D). In the stainless-steel wines, there was an increase in the “red fruit” and “dark fruit” attributes at the low dilution percentages.

**Fig. 12:**
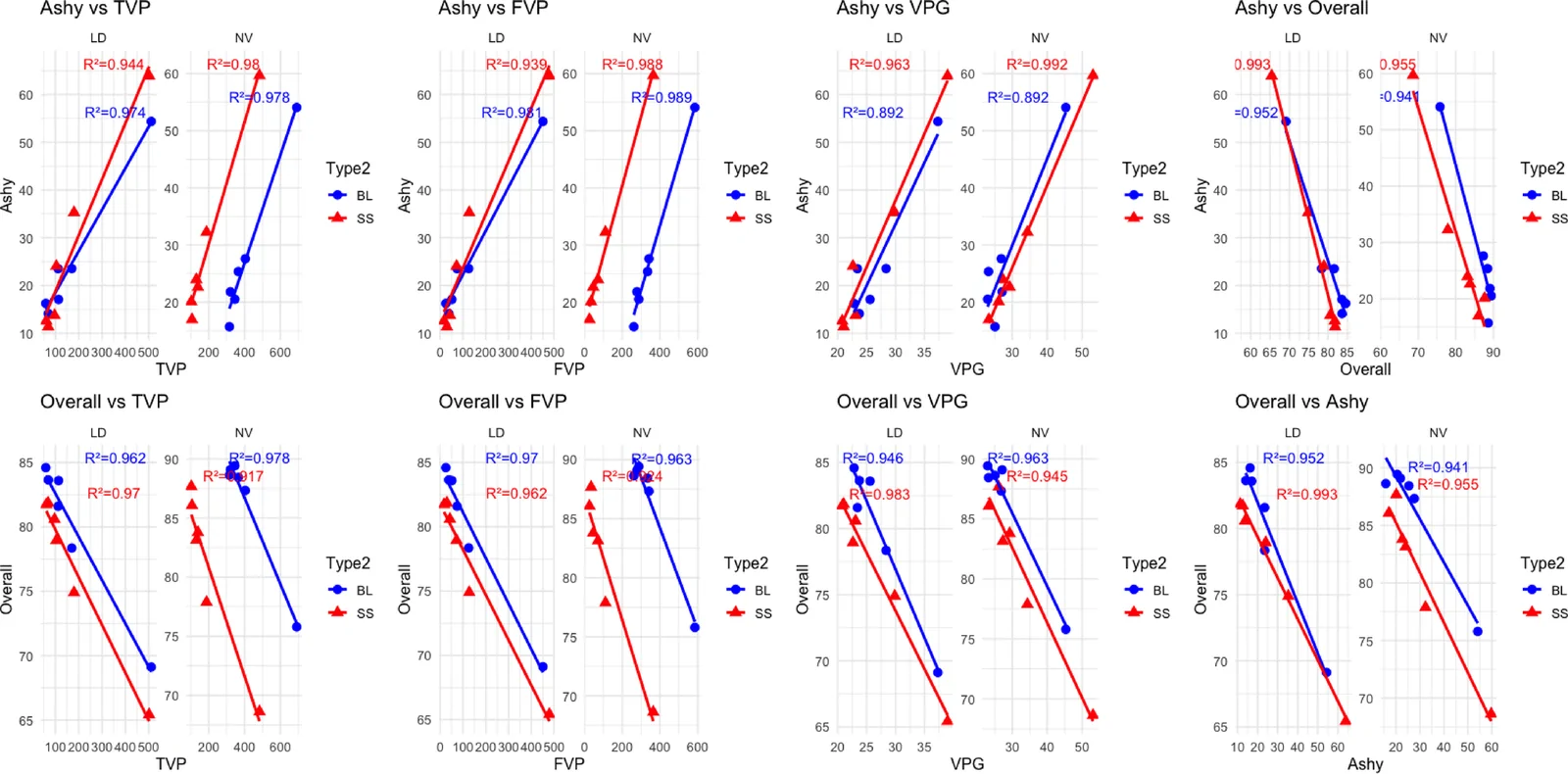
Correlation plot for each set of wines (Napa and Lodi) and each treatment (barrel-aged vs stainless steel) relating sum of TVPs, sum of FVPs, sum of VPGs, ashy score, and overall quality score. Regressions were performed separately for each wine set and treatment using six mean values corresponding to L0, L2, L3, L4, L5, and ST. The reported R^2^ values represent descriptive within-series relationships across ordered smoke-impact dilution levels rather than independent predictive models.

In the correlation and regression analysis (Fig. 12), scatter plots were generated for the following relationships: ashy versus the sum of TVP, FVP, and VPG, as well as ashy versus the overall quality score. Similarly, the overall quality score was plotted against the sum of TVP, FVP, and VPG, and against ashy. Regressions were conducted separately for each wine origin and aging treatment using six mean values corresponding to L0, L2, L3, L4, L5, and ST. Strong positive relationships were observed between ashy intensity and the summed chemical markers TVP, FVP, and VPG, with R^2^ values ranging from 0.892 to 0.992, while ashy and overall quality score were negatively correlated (R^2^ = 0.941–0.993). In the overall category, negative correlations were also found between overall quality score and the sum of FVP, TVP, VPG, and ashy (R^2^ = 0.917–0.993). All Pearson’s r values were significant at *p* < 0.05. However, because these regressions were based on ordered dilution levels, the high R^2^ values should be interpreted as descriptive of the dilution series rather than independent predictive relationships.

## Discussion

Oak barrel aging increased the concentrations of volatile phenols and volatile phenol glycosides, but at the same time lowered the perceived intensities of smoke taint sensory attributes and improved the perceived quality of the smoke-affected wines (Figs. 4 and 6). Barrel-aged wines had lower smoky attribute intensities for “ashy”, “cigarette smoke”, “liquid smoke” and “sweet BBQ”, when compared to their stainless-steel counterparts. The range of the VPs and VPGs concentrations we measured was consistent with that found in previous studies on overall smoke exposure^10,61,62^. In barrel-aged wines, there was an increase in the concentration of volatile phenols and their respective glycosides, with some concentrations exceeding the threshold perception levels^5,6^. This was seen in the Napa wine for guaiacol and methyl guaiacol concentrations, which are smoky and campfire-like in smell. Total VPs increased for the barrel treatments, yet the intensities of their aromas were lower in the barrel-aged wines. This suggests that the measured concentrations of VPs and VPGs alone did not determine smoke-related sensory perception in the barrel-aged wines. Instead, the lower perceived smoke intensity may reflect perceptual mitigation, where smoke-related attributes were suppressed or masked within the more complex oak-aged wine matrices. Similarly, we also observed increases in the concentrations of *p*-cresol, which is described as tar and medicinal, in the barrel-aged wines compared to the stainless-steel wines. This could be attributed to the varying contributions of the glycosides, which add more to the barrel impact than the overall smoke impact^5,33^. This study did not analyze other oak-related volatiles, such as vanillin, aldehydes, and lactones, which are also known contributors to oak barrel aromas, and could have masked the smoke-related aromas^63^. Other matrix-driven mechanisms may also have contributed, including changes in phenolic structure during barrel aging, tannin-phenol or polysaccharide-aroma interactions, altered oral or retronasal release of volatile phenols, and possible adsorption of some smoke-related compounds to oak surfaces. However, these mechanisms were not directly measured in the present study and therefore remain hypotheses.

As such, the proposed sensory masking or suppression effect cannot be chemically verified in the present study. This is an important limitation, as toasted oak can also contribute volatile phenols such as guaiacol, cresols, and syringol, which overlap with compounds commonly associated with smoke exposure. The increased volatile phenol concentrations observed in barrel-aged wines may reflect contributions from both smoke exposure and oak contact, while the reduced smoke perception may result from sensory interactions with unmeasured oak-derived aroma compounds. Therefore, the mitigation observed here should be interpreted primarily as a reduction in perceived smoke-related sensory intensity under the conditions tested, rather than as direct chemical evidence of smoke-taint removal or transformation. Future studies should include analysis of known oak marker compounds such as vanillin, furfural, 5-methylfurfural, oak lactones, eugenol, syringaldehyde, coniferaldehyde, and ellagitannin-related markers, to better distinguish oak contribution from smoke-derived phenolics and to confirm the role of sensory masking.

The blending of a small amount of smoke-tainted wine in the original wine, as in the high dilution and low smoke-taint L1 wines, added complexity to the wines, which in turn improved their perceived sensory quality. This was observed for both wine types (full-bodied Napa Cabernet Sauvignon and medium-bodied Lodi Cabernet Sauvignon) and treatments (stainless-steel wines and barrel-aged wines), where quality scores for these wines initially increased (but not significantly) with level of smoke taint before decreasing (Fig. 11). The wine blends with low levels of smoke impacted wine could be more complex from a sensory standpoint, and at the same time, their VP and VPG concentrations were also low, and may not have contributed towards the smoky characters of the wines^23,64^. This further supports the interpretation that the sensory impact of smoke-related compounds depends on the broader wine matrix and context, rather than on VP and VPG concentrations alone. There was a reduction in the “medicinal/brett” aroma intensity in the Lodi wines and reduction of 4-ethylphenol concentration after barrel aging which could have led to an increase in the quality of the wines. There was an increase in the overall quality score initially before decreasing. Fruit attributes, “cooked fruit”, “red fruit” are possible drivers of this increase in quality in the stainless-steel wine at low dilutions, yet this cannot be said of the barrel-aged wines, for which differences were only in smoke attributes in both Napa barrel-aged and Lodi barrel-aged wines, and in “vanilla” and “oaky” in the Lodi barrel-aged wines only.

A limitation of the study is that the L1 wines was excluded from the descriptive analysis, although it was included in the Wine Cuality™ assessment. This decision was made because bench testing indicated that L1 was highly similar to L0/NST, and its inclusion would have increased the sample load for trained panelists without adding clear sensory separation. Consequently, the PCA and MFA based on DA data represent L0/NST, L2–L5, and ST wines only, whereas the Wine Cuality™ quality maps include the complete dilution series. This difference in sample inclusion should be considered when comparing descriptive sensory profiles with expert quality assessments. The exclusion of L1 limits interpretation of the earliest transition from non-smoke-tainted wine to very low smoke-impact wine in the DA dataset, but it is unlikely to alter the broader interpretation of smoke-impact progression, barrel-aging effects, or the relationship between smoke-related sensory attributes and perceived wine quality.

The Wine Cuality^™^ method proved to be a useful alternative tool in evaluating the quality of both the stainless-steel and barrel-aged wines. It was important to provide some context for the experts prior to tasting the wines, i.e., that the first set of wines they tasted had only been processed in stainless steel and should therefore be judged on their promise (potential before aging), and that the second set of wines had been barrel-aged and were to be judged like any commercial Cabernet Sauvignon wine. Yet, wines were served blind without experts knowing the actual goal of the study; hence, doing a quality evaluation of the wine as a ready-for-market wine or the promise of a wine before it underwent aging allowed for an independent evaluation of these wines to better understand their quality and if the faults (smoke-taint) did indeed penalize the wine itself. The Wine Cuality scores were related to the descriptive attribute intensities to give an accurate description of the impact of smoke exposure at various levels and to show the overall negative linear impact on the quality of the wines. This is in line with work done previously by Parker and collaborators, and Tan and collaborators, who saw an overall linear relationship between smoke exposure and perceived quality^61,65^. Based on Fig. 11, there was an observed negative linear relationship whereby the drop in quality scores was negatively correlated with the increase in smoky/ashy descriptive attribute intensities. A drop in quality of more than 10 points on the 100-point scale is considered significant in the Wine Cuality method^46^. Using this difference threshold, in the Napa wines, such a quality drop was observed between wines L5 (25% smoked wine) and ST for both stainless-steel and barrel-aged wines. The drop was larger in the stainless-steel as compared to the barrel-aged Napa wines. For the Lodi wines, such a quality drop was observed between wines L4 (12.5% smoked wine) and L5 (25% smoked wine) for both the stainless-steel and the barrel-aged wines. This could be the result of differences between the matrices of the Napa and Lodi wines and the perception of quality in them. These findings may help winemakers estimate the proportion of smoke-affected wine that can be blended while still achieving a wine of acceptable quality.

Barrel-aged wines consistently received higher quality ratings for both sets of wines which was as expected as these wines were more akin to commercial oak-aged Cabernet Sauvignon wines. How a particular variety and style of wine should taste and smell is indeed fairly well defined both in industry circles and in the many wine education and appreciation programs that exist, such as the Court of Master’s Sommeliers, Institute of Master of Wine, and Wine & Spirit Education Trust (WSET) programs, where each wine style is carefully defined^66–68^. However, these higher quality ratings should not be interpreted solely as evidence of smoke-taint reduction. The barrel-aged wines were evaluated as finished commercial Cabernet Sauvignon wines, whereas the stainless-steel wines were evaluated for their promise before aging. Therefore, the higher Wine Cuality™ scores for barrel-aged wines likely reflect a combination of reduced smoke-related attribute intensities, improved overall sensory quality, and greater conformity to expected commercial oak-aged Cabernet Sauvignon style. In this context, DA provides evidence for lower intensities of specific smoke attributes, while Wine Cuality™ reflects broader expert quality judgment within the relevant wine style.

Of (sensory evaluation methodology) note, the percentage of the variance accounted for in the correspondence analysis of the CATA selections by the experts was similar to that accounted for in the PCA of the descriptive analysis ratings for both Lodi wines (84.4% vs. 85.9%) and Napa wines (86.2% vs. 88.9%), which points to an equally adequate characterization of the sensory properties of a set of wines by descriptive analysis compared to CATA (even by experts), and to the greater sensory complexity of the Napa Cabernet Sauvignon wines, which required more contrasting dimensions to adequately describe them.

The impact of smoke on the wine viticultural area and style (full-bodied, Napa vs medium-bodied, Lodi) was observed mainly in the quality scores. The mean quality ratings displayed in Fig. 6 confirmed the higher quality of the Napa Cabernet Sauvignon compared to that of the Lodi Cabernet Sauvignon, with quality ratings ranging from 88.63 to 75.79 for the barrel-aged Napa CS vs. 84.6 to 69.12 for the barrel-aged Lodi CS, and 86.07 to 68.63 for the stainless-steel Napa CS vs. 81.74 to 65.43 for the stainless-steel Lodi CS. Yet, it is important to note that when these wines are clustered based on the quality scores (Fig. 7), the point where the dip in quality is observed is consistent across viticultural areas for each treatment type.

The benefits of barrel aging were evident in both wine styles (Napa and Lodi), with oak-aged wines receiving higher quality scores compared to their stainless-steel counterparts. This improvement can be attributed to oak-derived characters such as “vanilla” and “oaky,” which likely enhanced palatability and overall sensory appeal^69^. In this research, it was observed that barrel aging reduced the perception of smoke-related attributes, including “ashy,” “cigarette smoke,” “liquid smoke,” and “sweet BBQ” across both wine styles, and additionally “tar” and “medicinal/brett” in the Napa wines at the L5 and ST taint levels. The co-increase of “cigarette smoke,” “liquid smoke,” and “sweet BBQ” with “ashy” confirmed the latter’s suitability as the primary sensory marker of smoke taint.

Across all correlation and regression analyses relating smoke sensory attributes and quality to the concentrations of relevant smoke taint markers, except for ashy vs. VPG in the Napa wines, barrel-aged wines contained higher concentrations of FVPs, TVPs, and VPGs than stainless-steel wines, yet exhibited lower ashy ratings and higher overall scores. For the Napa wines ashy vs. VPG regression, stainless-steel wines had higher VPG concentrations than barrel-aged wines, which received higher ashy ratings. It could also indicate that there are compounds causing the ashy character that still are not being measured. Collectively, these results demonstrate that overall quality and ashy are inversely related: an increase in ashy intensity consistently leads to a decrease in the overall quality score for the wine. And they validate the ashy descriptor and standard as a good sensory marker of smoke taint in red wine.

Regression and correlation analysis also demonstrated the clear impact of barrel aging on wine quality: despite increases in the chemical markers VPG, FVP, and TVP in barrel-aged wines, the intensity of the “ashy” character decreased, and overall quality scores improved. This indicates that while oak contributes compounds associated with these volatile phenols, they do not enhance smoke taint perception. Interestingly, at low smoke-taint levels (wines L0 and L2), barrel-aged wines displayed slightly higher “ashy” intensities compared to stainless-steel wines, suggesting that toasty barrel notes may have overlapped with, and been perceived as mildly “ashy”, yet contributed positively to wine complexity and quality^10,43,61,62^. These relationships should be interpreted cautiously because each regression was based on six mean values from an ordered dilution series, L0, L2, L3, L4, L5, and ST. Therefore, the high R^2^ values likely reflect, at least in part, the structured increase in smoke-affected wine across dilution levels, rather than independent biological or chemical observations. These analyses are best interpreted as descriptive evidence of how smoke-related chemistry, ashy perception, and perceived quality changed across the smoke-impact gradient within each wine and treatment. Within this limitation, barrel aging increased wine quality by amplifying desirable oak-related attributes while masking or reducing the perception of smoke-derived sensory faults at higher taint concentrations. It may also contribute to the removal of some smoke related compounds that cause the ashy character. This supports the hypothesis that oak-derived sensory attributes may have reduced or masked the perception of smoke-related faults. However, the underlying mechanisms, including possible masking effects and adsorption to oak, remain hypotheses as they were not directly measured in this study, and further work is required to confirm these mechanistic processes. An additional risk of the use of an oak barrel is the potential smoke carry over effect into subsequent use of that barrel.

This study demonstrated the potential mitigating effect of oak barrel aging on smoke perception in Cabernet Sauvignon under the specific conditions tested. However, these findings are limited to Cabernet Sauvignon wines from two California regions, a post-harvest smoke exposure system, selected smoke-impact dilution levels, single winemaking batches of wine, and 12 months of aging in the oak barrels used in this study. Therefore, the results should not be generalized to all cultivars, wine styles, smoke exposure timings, smoke fuel sources, oak toast levels, barrel ages, oak alternatives, or commercial wildfire-exposed wines without further validation. Future research should explore the effectiveness of oak mitigation in other red and white varieties, different smoke exposure conditions, and a wider range of oak treatments, including barrels of different ages and toast levels, as well as oak shavings, chips, staves, dust, and extracts, and ideally, through multiple winemaking replications. There would also be a potential risk of carryover effects when smoke-tainted wines are aged in oak barrels, particularly in new barrels, which may impact the untainted wine that is subsequently aged in such a barrel. This highlights practical considerations for the reuse of barrels and suggests that alternative oak treatments should be further investigated as well. This stresses the need to evaluate both mitigation efficacy and practical cellar-management risks before broader application.

## Conclusion

The use of oak barrel aging to enhance the sensory quality of wine has been well established. However, its potential role in mitigating smoke taint had not been systematically researched or applied commercially, largely due to the high cost of barrels and the risk of smoky flavor carryover. Smoke impact manifests at two levels: a moderate reduction in quality at low concentrations, and a distinct taint at higher concentrations. Under the conditions tested in this study, oak barrel aging reduced the perceived intensity of smoke-related attributes and improved perceived wine quality in Cabernet Sauvignon wines from two California regions.

These findings suggest that oak barrel aging may have mitigation potential for some smoke-affected Cabernet Sauvignon wines, particularly when smoke impact is moderate and when the resulting wine remains consistent with the intended commercial style. However, this conclusion is limited to the specific wines, smoke exposure system, dilution levels, barrel treatment, and 12-month aging period evaluated here. The results should therefore not be interpreted as evidence that oak aging is broadly effective for all smoke-affected wines or wine styles. Future work should evaluate other cultivars, smoke exposure extent and sources, oak toast levels, barrel ages, oak alternatives, and commercially wildfire-exposed wines.

Given that many commercial Cabernet Sauvignon wines undergo some form of oak treatment, this approach may have practical relevance for winemakers seeking to improve complexity while reducing the perception of smoke-related faults. We recommend that wineries conduct bench-scale trials and sensory evaluations with a trained panel to assess whether affected wines remain salvageable and meet house style and quality standards before using this strategy.

Finally, we encourage the use of the Wine Cuality method as a robust and holistic framework for evaluating and documenting wine quality across the production process, offering a more comprehensive alternative to conventional expert quality assessments.

## Supplementary Information

Supplementary Information.

## Data Availability

The datasets used and/or analyzed during the current study is available from the corresponding authors on request.
