# Supplementary material for: Oak aging mitigates the sensory impact of smoke taint in Cabernet Sauvignon wine
Source: Sci Rep. 2026 Jun 30;16:27926. doi: 10.1038/s41598-026-59592-7 (PMC13550436; doi:10.1038/s41598-026-59592-7)
Supplement: Supplementary file 1 [file 41598_2026_59592_MOESM1_ESM.docx]

**Oak aging mitigates the sensory impact of smoke taint in Cabernet Sauvignon wine.**

Authors: Lik Xian Lim^1*^, Cristina Medina-Plaza^1^, Catherine Routt^1^, Reid Rodriguez^1^, Larry Lerno^1^, Jean-Xavier Guinard^2*^ and Anita Oberholster^1†^

^1^Department of Viticulture and Enology, University of California Davis, Davis, CA, 95616, USA

^2^Department of Food Science and Technology, University of California, Davis, Davis, CA, 95616, USA

*Corresponding author

^†^Deceased

Email (LXL): [lxlim@ucdavis.edu](mailto:lxlim@ucdavis.edu)

Email (JXG): [jxguinard@ucdavis.edu](mailto:jxguinard@ucdavis.edu)

**Supplementary Materials**

**Supplementary Table S1:** Descriptive analysis list of descriptors and preparation methods. The base wine used was Barefoot Pinot noir (Barefoot Cellars, Modesto, CA, USA).

| S/n | Category | Descriptor | Recipe | Brand |
| --- | --- | --- | --- | --- |
| 1 | Aroma | Cigarette smoke | Pinch of ash of loose leaf tobacco + 20 mL base wine | No. 91 Danish export Virginia blend cigarette tobacco. |
| 2 | Aroma | Musty | 2cm square wet cardboard + 5 mL water to wet evenly |  |
| 3 | Aroma | Medicinal / brett | 1 drop 40% phenol solution + 40 mL base wine |  |
| 4 | Aroma | Liquid smoke | 1 mL Wrights liquid smoke hickory with 40 mL base wine. | Wright's liquid smoke concentrated seasoning, hickory |
| 5 | Aroma | Sweet bbq | 1 mL Stubbs mesquite liquid smoke with 40 mL base wine. | Stubbs mesquite liquid smoke |
| 6 | Aroma | Menthol | 1 mL solution (3 drops eucalyptus oil in 100 ml water) + 40 mL base wine | Nature's Truth eucalyptus 100% pure essential oil |
| 7 | Aroma | Tar | 5mm slice of Bicycle inner tube with one corner in Asphalt cement | Bell 12.5" bicycle inner tube, black jack all weather roof cement |
| 8 | Aroma | Solvent | 0.125ml acetone, 1 drop ethyl acetate, 50ml base wine | Acetone- day logic brand 100% acetone |
| 9 | Aroma | Red fruit | 2 frozen dark cherry, 2 frozen raspberry, 1 frozen strawberry cut into cubes and smashed, 1 cm cube of the frozen strawberry. | Strawberry: Cascadian Farm (Sedro Woolley, WA)  Dark Cherry & Raspberry: Woodstock organic |
| 10 | Aroma | Dark fruit | 4 frozen blackberry, 2 dark frozen cherries, 5 frozen blueberry, all cut and smashed. + 1 drop Everclear | Blueberry: Cascadian Farm (Sedro Woolley, Wa), dark cherry: Woodstock organic, blackberries: Stahlbush Island Farms; Everclear, 120 proof |
| 11 | Aroma | Jammy fruit | 2 tsp of each blueberry jam, blackberry jam, 1 tsp strawberry and 1 raspberry jam in 20 mL red wine base | Wild blueberry jam- St. Dalfour brand, strawberry, blackberry, raspberry preserve- Smuckers |
| 12 | Aroma | Dried fruit | 1/8 pcs prune + 4 raisins + 1/4 dried figs +10 mL wine | Sunsweet d’noir prunes preservative free;  Sunmaid raisins; Sunmaid mission figs |
| 13 | Aroma | Bell pepper | 0.5 cm square piece of green bell pepper + wine 40 mL, 10 mins, (3 pcs of 2cmx2cm square in 600 mL wine, soak 10 mins, then filter |  |
| 14 | Aroma | Spice | 1 chip nutmeg + 1 chip cinnamon + 4 pcs all spice + 4pcs cloves, in 40 mL base wine, soak 50 mins and filter | Nutmeg: Morton and Barret,  Cinnamon, all spice, cloves: Mc Cormick |
| 15 | Aroma | Alcohol hotness | 16% alcohol final content, 300 mL 60% v/v Everclear | Everclear, 120 proof |
| 16 | Aroma | Vanilla | 0.250 mL vanilla extract, 50 mL base wine- (add wine first) | Mc Cormick gourmet organic premium pure vanilla extract |
| 17 | Aroma | Oaky | 1 tbsp feel wood sweet oak chips + 20 mL base wine (do night before) | Feel wood sweet oak chips |
| 18 | Mouthfeel/Taste | Sweet | Low 5g/L Fructose, High 12.5g/L Fructose in water | Now Real Food |
| 19 | Mouthfeel/Taste | Sour | Low 0.5g/L Tartaric Acid, High 2g/L Tartaric Acid in water |  |
| 20 | Mouthfeel/Taste | Bitter | Low- 500 mg/L Caffeine, High 1000 mg/L in water | Caffeine anhydrous, VWR Life Science |
| 21 | Mouthfeel/Taste | Dry | Low- 200 mg/L Alum, High 620 mg/L Alum in water | Mc Cormick |
| 22 | Mouthfeel/Taste | Hot | Low- 85mL/L 60% v/v Everclear, High- 170mL/L 60% v/v Everclear diluted with water | Everclear, 120 proof |
| 23 | Mouthfeel/Taste | Viscosity | Low- plain distilled Water, High 2g/L CMC in distilled water | Sugarman candy. |
| 24 | Ashy | Ashy aftertaste | Burnt leeks in water, concentrate, 1:5 for 100% solution, dilute solution 10% |  |

**Supplementary Table S2:** Basic chemical parameters for the Napa (NV) and Lodi (LD), stainless-steel and barrel-aged wines.

| Wine | Ethanol Content % (%v/v) | pH | Titratable Acidity (g/L) | Residual Sugar (g/L) | Malic Acid (mg/L) | Acetic Acid ( g/L) |
| --- | --- | --- | --- | --- | --- | --- |
| Stainless steel | | | | | | |
| NV_SS_L0 | 15.4 ± 0.0 | 3.6 ± 0.0 | 5.8 ± 0.0 | 0.4 ± 0.1 | 65.3 ± 19.7 | 0.3 ± 0.0 |
| NV_SS_L1 | 15.4 ± 0.0 | 3.7 ± 0.0 | 5.6 ± 0.0 | 0.3 ± 0.0 | 50.0 ± 0.0 | 0.4 ± 0.0 |
| NV_SS_L2 | 15.4 ± 0.0 | 3.6 ± 0.0 | 5.9 ± 0.2 | 0.5 ± 0.0 | 50.7 ± 5.5 | 0.3 ± 0.0 |
| NV_SS_L3 | 15.4 ± 0.0 | 3.7 ± 0.0 | 5.8 ± 0.1 | 0.5 ± 0.0 | 54.3 ± 3.8 | 0.3 ± 0.0 |
| NV_SS_L4 | 15.5 ± 0.0 | 3.7 ± 0.0 | 5.8 ± 0.1 | 0.5 ± 0.1 | 52.0 ± 13.5 | 0.3 ± 0.0 |
| NV_SS_L5 | 15.4 ± 0.0 | 3.7 ± 0.0 | 5.8 ± 0.1 | 0.5 ± 0.0 | 63.7 ± 14.5 | 0.3 ± 0.0 |
| NV_SS_ST | 15.7 ± 0.0 | 3.7 ± 0.0 | 5.7 ± 0.1 | 0.6 ± 0.0 | 80.3 ± 9.6 | 0.3 ± 0.0 |
| Barrel aged | | | | | | |
| NV_BL_L0 | 15.5 ± 0.0 | 3.6 ± 0.0 | 6.2 ± 0.0 | 0.4 ± 0.0 | 90.7 ± 20.1 | 0.6 ± 0.0 |
| NV_BL_L1 | 15.5 ± 0.0 | 3.6 ± 0.0 | 6.1 ± 0.0 | 0.4 ± 0.0 | 50.0 ± 0.0 | 0.6 ± 0.0 |
| NV_BL_L2 | 15.5 ± 0.1 | 3.6 ± 0.0 | 6.4 ± 0.1 | 0.5 ± 0.0 | 96.3 ± 17.4 | 0.6 ± 0.0 |
| NV_BL_L3 | 15.5 ± 0.1 | 3.6 ± 0.0 | 6.4 ± 0.0 | 0.4 ± 0.0 | 111.3 ± 3.5 | 0.6 ± 0.0 |
| NV_BL_L4 | 15.5 ± 0.0 | 3.6 ± 0.0 | 6.4 ± 0.1 | 0.5 ± 0.0 | 90.3 ± 20.7 | 0.6 ± 0.0 |
| NV_BL_L5 | 15.6 ± 0.0 | 3.6 ± 0.0 | 6.3 ± 0.0 | 0.4 ± 0.0 | 103.0 ± 11.8 | 0.6 ± 0.0 |
| NV_BL_ST | 15.9 ± 0.0 | 3.6 ± 0.0 | 6.3 ± 0.0 | 0.3 ± 0.1 | 110.0 ± 19.0 | 0.6 ± 0.0 |
| Stainless steel | | | | | | |
| LD_SS_L0 | 13.5 ± 0.0 | 3.8 ± 0.0 | 5.1 ± 0.1 | 0.3 ± 0.0 | 65.3 ± 42.3 | 0.3 ± 0.0 |
| LD_SS_L1 | 13.5 ± 0.0 | 3.8 ± 0.0 | 5.1 ± 0.0 | 0.1 ± 0.0 | 50.0 ± 0.0 | 0.3 ± 0.0 |
| LD_SS_L2 | 13.5 ± 0.0 | 3.8 ± 0.0 | 5.1 ± 0.0 | 0.2 ± 0.1 | 58.7 ± 33.2 | 0.3 ± 0.0 |
| LD_SS_L3 | 13.4 ± 0.0 | 3.8 ± 0.0 | 5.1 ± 0.0 | 0.2 ± 0.0 | 78.7 ± 17.0 | 0.3 ± 0.0 |
| LD_SS_L4 | 13.3 ± 0.0 | 3.8 ± 0.0 | 5.1 ± 0.0 | 0.2 ± 0.1 | 64.7 ± 17.4 | 0.4 ± 0.1 |
| LD_SS_L5 | 13.3 ± 0.0 | 3.8 ± 0.0 | 5.1 ± 0.0 | 0.2 ± 0.0 | 71.3 ± 11.5 | 0.4 ± 0.1 |
| LD_SS_ST | 12.5 ± 0.0 | 3.9 ± 0.0 | 5.0 ± 0.1 | 0.3 ± 0.0 | 94.0 ± 31.2 | 0.4 ± 0.1 |
| Barrel aged | | | | | | |
| LD_BL_L0 | 13.6 ± 0.1 | 3.7 ± 0.0 | 5.5 ± 0.1 | 0.2 ± 0.2 | 90.3 ± 49.1 | 0.4 ± 0.0 |
| LD_BL_L1 | 13.6 ± 0.0 | 3.8 ± 0.0 | 5.4 ± 0.0 | 0.1 ± 0.0 | 50.0 ± 0.0 | 0.5 ± 0.0 |
| LD_BL_L2 | 13.6 ± 0.0 | 3.7 ± 0.0 | 5.6 ± 0.1 | 0.2 ± 0.0 | 62.7 ± 44.1 | 0.4 ± 0.0 |
| LD_BL_L3 | 13.5 ± 0.0 | 3.7 ± 0.0 | 5.6 ± 0.1 | 0.2 ± 0.0 | 56.3 ± 32.1 | 0.4 ± 0.0 |
| LD_BL_L4 | 13.5 ± 0.0 | 3.7 ± 0.0 | 5.6 ± 0.1 | 0.2 ± 0.1 | 78.0 ± 19.7 | 0.4 ± 0.0 |
| LD_BL_L5 | 13.3 ± 0.0 | 3.7 ± 0.0 | 5.6 ± 0.1 | 0.2 ± 0.1 | 81.7 ± 2.1 | 0.5 ± 0.0 |
| LD_BL_ST | 12.7 ± 0.0 | 3.8 ± 0.0 | 5.4 ± 0.0 | 0.2 ± 0.1 | 86.7 ± 54.2 | 0.5 ± 0.0 |

**Supplementary Table S3**: Significant sensory attribute overall means with Fisher’s LSD for the Napa Cabernet Sauvignon wines on a scale between 0 to 100 points.

| Wine | Red.Fruit | Cooked.Fruit | Vanilla | Cigarette.Smoke | Liquid.Smoke | Sweet.BBQ | Solvent | Tar | Ashy |
| --- | --- | --- | --- | --- | --- | --- | --- | --- | --- |
| Stainless Steel | | | | | | | | | |
| NV_SS_L0 | 28.03 ab | 35.23 a | 25.27 a | 11.20 b | 7.83 b | 6.40 b | 23.80 a | 6.00 b | 16.97 c |
| NV_SS_L2 | 28.46 ab | 36.87 a | 20.70 ab | 12.33 b | 6.23 b | 6.93 b | 18.63 ab | 3.27 b | 20.10 c |
| NV_SS_L3 | 28.80 a | 38.20 a | 21.40 ab | 14.83 b | 9.80 b | 7.90 b | 17.17 ab | 6.00 b | 22.63 bc |
| NV_SS_L4 | 30.20 a | 32.47 a | 20.40 ab | 15.27 b | 8.03 b | 6.13 b | 15.60 ab | 4.43 b | 23.97 bc |
| NV_SS_L5 | 24.73 ab | 36.30 a | 23.40 ab | 17.40 b | 11.70 b | 11.07 b | 19.73 ab | 8.67 ab | 32.27 b |
| NV_SS_ST | 18.50 b | 29.33 a | 14.60 b | 30.23 a | 28.40 a | 28.53 a | 12.67 b | 13.13 a | 59.70 a |
|  | | | | | | | | | |
| Wine | Red.Fruit* | Cooked.Fruit* | Vanilla* | Cigarette.Smoke | Liquid.Smoke | Sweet.BBQ | Solvent* | Tar* | Ashy |
| Barrel | | | | | | | | | |
| NV_BL_L0 | 27.46 | 30.46 | 26.85 | 13.55 bc | 7.55 b | 7.73 b | 22.667 | 6.49 | 15.67 c |
| NV_BL_L2 | 25.06 | 33.15 | 25.21 | 7.52 c | 6.36 b | 7.94 b | 20.909 | 6.39 | 21.82 bc |
| NV_BL_L3 | 29.24 | 29.18 | 25.15 | 11.57 bc | 8.12 b | 8.12 b | 24.515 | 8.18 | 20.52 bc |
| NV_BL_L4 | 25.49 | 30.49 | 25.49 | 16.42 ab | 10.97 ab | 10.03 b | 21.788 | 9.39 | 25.36 b |
| NV_BL_L5 | 20.64 | 27.73 | 22.91 | 14.30 abc | 11.33 ab | 11.79 b | 22.667 | 8.64 | 27.61 b |
| NV_BL_ST | 23.42 | 28.33 | 22.85 | 21.52 a | 14.39 a | 22.27 a | 18.909 | 10.85 | 54.09 a |

*The attribute was not significant for the specific treatment..

**Supplementary Table S4:** Significant sensory attribute overall means with Fisher’s LSD for the Lodi Cabernet Sauvignon wines on a scale between 0 to 100 points.

| Wine | Red Fruit | Dark Fruit | Cooked Fruit | Spice | Vanilla | Oaky* | Cigarette Smoke | Medicinal Brett | Liquid Smoke | Sweet BBQ | Tar | Ashy |
| --- | --- | --- | --- | --- | --- | --- | --- | --- | --- | --- | --- | --- |
| Stainless Steel | | | | | | | | | | | | |
| LD_SS_L0 | 31.90 a | 27.93 ab | 31.50 a | 15.30 a | 21.27 ab | 20.20 a | 9.87 bc | 21.03 ab | 3.73 c | 4.43 b | 7.80 b | 12.60 d |
| LD_SS_L2 | 29.30 ab | 31.93 a | 28.63 ab | 16.83 a | 21.73 a | 19.43 a | 9.50 c | 21.37 ab | 4.73 c | 5.70 b | 4.93 b | 11.37 d |
| LD_SS_L3 | 28.93 ab | 36.23 a | 30.27 ab | 14.07 a | 21.90 a | 23.63 a | 8.07 c | 18.80 b | 3.90 c | 4.80 b | 6.43 b | 13.77 d |
| LD_SS_L4 | 28.93 ab | 27.30 ab | 30.33 ab | 19.50 a | 23.17 a | 23.20 a | 14.20 bc | 27.83 ab | 9.50 bc | 9.67 b | 7.00 b | 24.07 c |
| LD_SS_L5 | 20.67 bc | 29.00 ab | 26.20 ab | 12.87 a | 18.00 ab | 20.77 a | 17.77 b | 28.93 a | 16.30 b | 11.60 b | 10.00 ab | 35.30 b |
| LD_SS_ST | 18.83 c | 20.00 b | 20.23 b | 12.17 a | 12.70 b | 19.20 a | 28.57 a | 29.93 a | 31.60 a | 31.30 a | 13.77 a | 63.93 a |
|  | | | | | | | | | | | | |
| Wine | Red.Fruit | Dark  Fruit* | Cooked.  Fruit* | Spice | Vanilla | Oaky | Cigarette  Smoke | Medicinal Brett | Liquid  Smoke | Sweet  BBQ | Tar | Ashy |
| Barrel | | | | | | | | | | | | |
| LD_BL_L0 | 22.28 a | 28.83 a | 28.64 a | 16.61 a | 21.00 a | 44.64 a | 9.36 b | 18.31 a | 7.92 b | 5.611 b | 7.36 b | 16.25 bc |
| LD_BL_L2 | 20.67 a | 29.17 a | 22.25 a | 17.17 a | 23.42 a | 41.75 ab | 9.39 b | 16.86 a | 6.11 b | 6.111 b | 6.31 b | 14.11 c |
| LD_BL_L3 | 24.33 a | 32.25 a | 26.11 a | 16.61 a | 24.19 a | 43.61 a | 9.69 b | 14.97 a | 7.97 b | 4.806 b | 6.33 b | 17.08 bc |
| LD_BL_L4 | 23.31 a | 29.69 a | 26.11 a | 16.39 a | 19.22 a | 47.33 a | 13.47 b | 15.75 a | 8.19 b | 7.972 b | 6.72 b | 23.53 b |
| LD_BL_L5 | 20.83 a | 30.44 a | 25.08 a | 17.94 a | 23.19 a | 44.72 a | 13.36 b | 18.50 a | 9.94 b | 8.722 b | 8.53 b | 23.56 b |
| LD_BL_ST | 16.64 a | 25.56 a | 23.83 a | 11.78 a | 16.56 a | 34.28 b | 28.50 a | 22.06 a | 24.11 a | 17.667 a | 14.44 a | 54.36 a |

* The attribute was not significant for the specific treatment.
